# Atlantic salmon populations invaded by farmed escapees: quantifying genetic introgression with a Bayesian approach and SNPs

**DOI:** 10.1186/1471-2156-14-74

**Published:** 2013-08-23

**Authors:** Kevin Alan Glover, Cino Pertoldi, Francois Besnier, Vidar Wennevik, Matthew Kent, Øystein Skaala

**Affiliations:** 1 Section of Population Genetics and Ecology, Institute of Marine Research, Bergen, Norway; 2 Department 18/Section of Environmental Engineering, Aalborg University, Sohngårdsholmsvej 57, Aalborg 9000, Denmark; 3 Department of Biosciences, Aarhus University, Ny Munkgade 114, Aarhus 820, Denmark; 4 Center for Integrative Genetics, Department of Animal and Aquacultural Sciences, Norwegian University of Life Sciences, Ås, Norway

**Keywords:** Admixture, Aquaculture, Environmental impact, Escapees, Simulation, ABC, Fish farming, Migration

## Abstract

**Background:**

Many native Atlantic salmon populations have been invaded by domesticated escapees for three decades or longer. However, thus far, the cumulative level of gene-flow that has occurred from farmed to wild salmon has not been reported for any native Atlantic salmon population. The aim of the present study was to investigate temporal genetic stability in native populations, and, quantify gene-flow from farmed salmon that caused genetic changes where they were observed. This was achieved by genotyping historical and contemporary samples from 20 populations covering all of Norway with recently identified single nucleotide polymorphism markers that are collectively diagnostic for farmed and wild salmon. These analyses were combined with analysis of farmed salmon and implementation of Approximate Bayesian computation based simulations.

**Results:**

Five of the populations displayed statistically significant temporal genetic changes. All five of these populations became more similar to a pool of farmed fish with time, strongly suggesting introgression of farmed fish as the primary cause. The remaining 15 populations displayed weak or non-significant temporal genetic changes. Estimated introgression of farmed fish ranged from 2-47% per population using approximate Bayesian computation. Thus, some populations exhibited high degrees of farmed salmon introgression while others were more or less unaffected. The observed frequency of escapees in each population was moderately correlated with estimated introgression per population R^2^ = 0.47 *P* < 0.001. Genetic isolation by distance existed within the historical and contemporary data sets, however, the among-population level of divergence decreased with time.

**Conclusions:**

This is the first study to quantify cumulative introgression of farmed salmon in any native Atlantic salmon population. The estimations demonstrate that the level of introgression has been population-specific, and that the level of introgression is not solely predicted by the frequency of escapees observed in the population. However, some populations have been strongly admixed with farmed salmon, and these data provide policy makers with unique information to address this situation.

## Background

Aquaculture production of Atlantic salmon (*Salmo salar* L.) was started in Norway in the early 1970′s, and now represents a globally significant industry. Each year, hundreds of thousands of farmed salmon escape into the wild [1]. Some of these escapees enter rivers inhabited by native populations [2-4], outnumbering wild conspecifics on the spawning grounds of some rivers in some years [5]. The Atlantic salmon is characterized by highly significant population genetic structuring [6,7]. This reflects evolutionary relationships among populations [8-10], including the potential for adaptive differences [11,12]. Consequently, the large-scale invasion of Atlantic salmon populations by domesticated farmed escapees represents one of the most striking examples of human-mediated increased straying rates for any organism. This has raised global concerns for the fitness of native populations [13-15].

Genetic changes in native Atlantic salmon populations as a result of introgression of farmed escapees have been observed in populations in Ireland [16-19], North America [20], and Norway [21,22]. In the most extensive of these studies, six of 21 Norwegian populations investigated displayed significant temporal genetic changes. Based upon several genetic parameters, the authors concluded that the observed changes were primarily driven by introgression of escapees. However, in none of the above-mentioned studies has the accumulated level of introgression, i.e., “admixture”, been quantified in a native population. From a management perspective, this is important, if not essential, in order to understand the extent of the problems, and ultimately implement guidelines via the process of risk assessment [23].

Where gene flow arises from a single and definable population or hatchery strain, statistical parameters such as individual-based admixture can be computed to estimate the level of introgression and degree of remaining wild population e.g., [24,25]. Even in cases of low numbers of populations, it is also possible to infer admixture using a combination of molecular genetic data on real samples in addition to simulations [26,27]. However, the quantification of genetic introgression of farmed Atlantic salmon into native Norwegian populations represents a more complicated situation than one in which a single or a low number of populations are exchanging genes among themselves [28]. This is because of several factors which are addressed briefly below.

The commercial production of Atlantic salmon in Norway is based upon rearing fish from multiple domesticated strains that were initially founded on wild salmon from more than 40 Norwegian rivers in the 1970′s [29]. These domesticated strains have remained genetically isolated from wild salmon since. As a result of founder effects and genetic drift, there are highly significant differences in microsatellite allele frequencies among these farmed strains [30]. Thus, microsatellites provide enough information to distinguish some farmed strains and wild populations in a pair-wise manner [30]. However, the allele frequencies of microsatellites [30] and SNPs [31] display overlap between the farmed strains and wild populations when looking across multiple strains and populations simultaneously. Over time, escapees originate from multiple farms. As a result, the accumulated genetic change in the native population, due to introgression of this pool of farmed salmon, becomes very complicated to quantify, and is potentially underestimated [28]. Adding further to the complexity of this situation is the fact that the domesticated strains have changed greatly over time. Some strains have been terminated, while others have been mixed. Thus, when these points are taken together with the fact that there is non-random distribution of genetic material from the breeding companies to the production farms [32] where the majority of the farmed salmon are held and thus the majority of escapees originate from, it is impossible to accurately reconstruct the allele frequencies of the farmed strains used in Norway in the three to four decade period in which escapees have been observed on the spawning grounds.

A recent genome scan using a 7 k chip identified a set of SNPs that are collectively diagnostic in identifying Norwegian wild and farmed salmon, regardless of their population of origin [31]. These collectively diagnostic markers have the potential to circumvent some of the challenges described above to quantify introgression of farmed salmon in native Norwegian populations. At the same time, statistical approaches such as Approximate Bayesian Computation (ABC) [33] have been used to quantify complex models in population and evolutionary biology [34-36]. The present study aimed to take advantage of these recently discovered genetic markers and genotype a set of historical and contemporary samples from 20 Norwegian salmon populations that have displayed varying level of farmed escapees on the spawning grounds over the past 2–3 decades. In addition, a pool of farmed salmon was genotyped in order to investigate the direction of any observed temporal genetic changes in the wild populations. Finally, ABC and fixed migration simulation methods were implemented in order to attempt to quantify the level of cumulative introgression of farmed salmon in native populations for the first time.

## Methods

### Wild salmon samples

**Figure 1:**
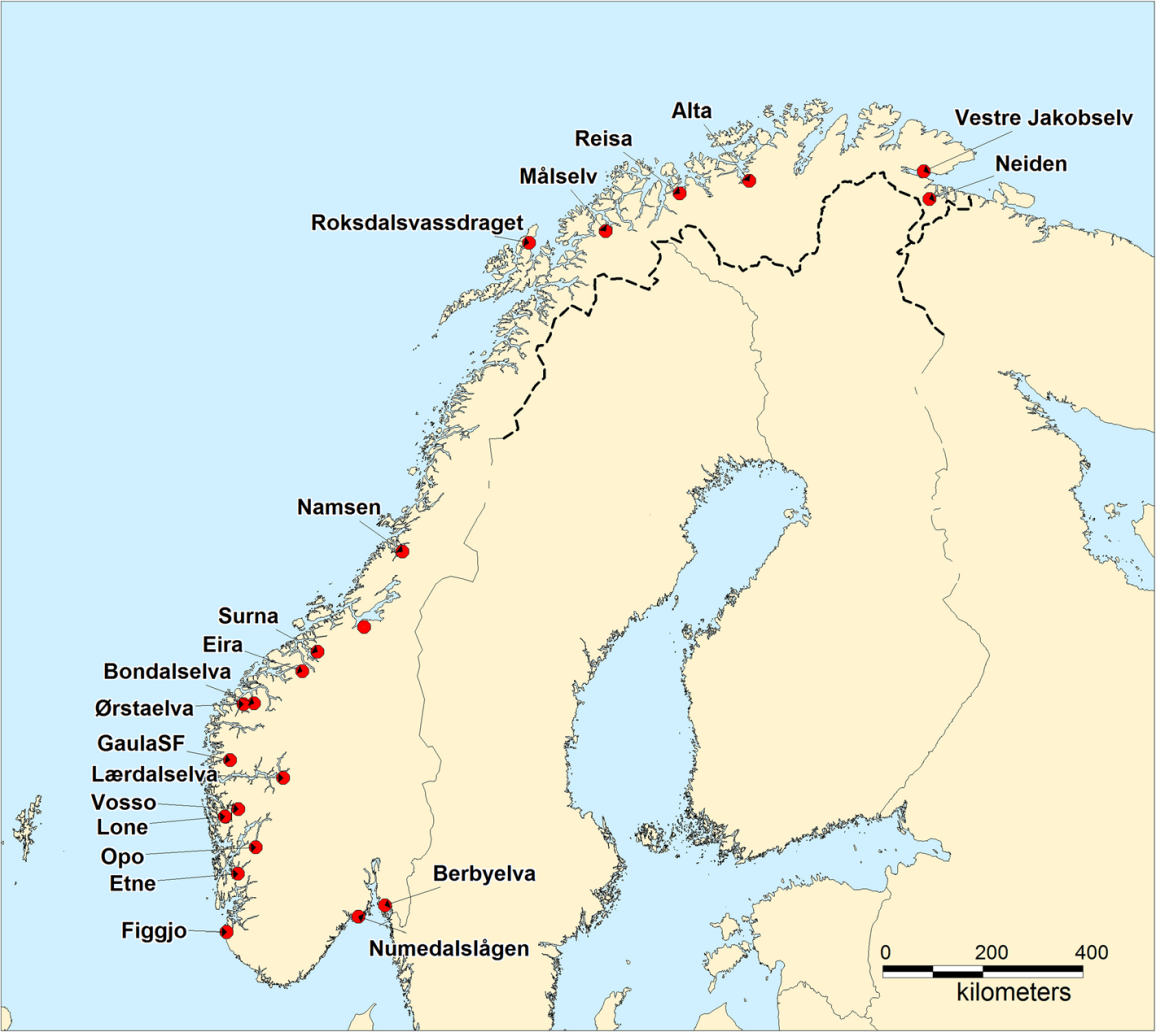
Location of the 20 rivers upon which the study is based.

Samples of wild salmon were collected from a total of 20 rivers spanning the entire coastline of Norway (Figure 1). Each river was represented by a historical sample that was collected prior to or in the early to moderate stages of the development of the commercial aquaculture industry in Norway, in addition to a contemporary sample that was collected in the period 2000–2009. Most of these samples were based upon fish scales taken from adult fish captured within each river (Table 1). The historical adult samples were primarily collected by the Norwegian Atlantic salmon gene bank. These samples were taken from multiple locations and years within each river to ensure a representative sample of each population. Of the contemporary adult samples, the majority were taken in association with recreational angling. These samples form the basis of the national monitoring program for estimating the frequencies of escapees within Norwegian rivers [1,4,5]. Where angling was the primary sampling method, samples were collected from multiple years and locations within each river in order to ensure the samples were as representative for the populations as possible. A few of the river samples were represented by juveniles (parr) collected by electrofishing (Table 1). These samples were collected in multiple locations within each river, and consisted of fish of varying age in order to ensure representative sampling. Due to the fact that the samples upon which this project were based were captured by recreational angling or in association with other previous research projects and monitoring programs (and subsequently donated to this study), no specific licenses were required for this specific study.

**Table 1:** Numbers and types of samples, including some population genetics summary statistics for the 20 Atlantic salmon rivers

**Population**	**N**	**uH**_**E**_	**HWE**	**LD**	**Sample type**	**Population**	**N**	**uH**_**E**_	**HWE**	**LD**	**Sample type**
Neiden H (1979–82)	70	0.35	2	130	AD	Ørsta H (1986–89)	38	0.38	2	98	AD
Neiden C (2009)	77	0.36	5	112	AD	Ørsta C (2006–08)	31	0.36	4	130	AD
V. Jakobselva H (1989–91)	92	0.35	5	243	AD	GaulaSF H (1987–93)	35	0.36	0	120	AD
V. Jakobselva C (2007–08)	96	0.37	2	183	AD	GaulaSF C (2006–08)	82	0.36	2	131	AD
Alta H (1988–90)	39	0.34	1	85	AD	Lærdalselva H (1973)	90	0.36	1	125	AD
Alta C (2005–2007)	63	0.34	2	102	P	Lærdalselva C (2005–08)	45	0.36	1	120	AD
Reisa H (1986–91)	44	0.35	4	101	AD	Vosso H (1980)	45	0.34	0	98	AD
Reisa C (2006)	55	0.35	1	136	P	Vosso C (2007–08)	43	0.36	0	138	SM
Målselva H (1986–88)	39	0.35	3	102	AD	Loneelva H (1986–93)	59	0.34	0	136	AD
Målselva C (2008)	30	0.36	1	111	P	Loneelva C (2001–07)	50	0.36	3	134	AD
Roksdalsvassdraget H (1987–93)	31	0.37	0	110	AD	Opo H (1971–73)	60	0.35	3	116	AD
Roksdalsvassdraget C (2008)	89	0.37	3	128	AD	Opo C (2010)	61	0.36	3	180	P
Namsen H (1977)	74	0.36	0	129	AD	Etne H (1983)	72	0.35	1	121	AD
Namsen C (2008)	89	0.37	1	140	AD	Etne C (2006–2008)	83	0.36	1	122	AD
Surna H (1986–89)	23	0.36	2	90	AD	Figgjo H (1972–75)	51	0.35	1	118	AD
Surna C (2005–08)	45	0.37	4	122	AD	Figgjo C (2006)	71	0.36	1	119	AD
Eira H (1986–94)	31	0.36	2	108	AD	Numedalslågen H (1989–93)	42	0.35	1	89	AD
Eira C (2005–2008)	40	0.35	1	123	AD	Numedalslågen C (2007–08)	68	0.36	3	132	AD
Bondalselva H (1986–88)	39	0.37	3	103	AD	Berbyelva H (1988–93)	44	0.33	1	132	AD
Bondalselva C (2007)	13	0.36	1	70	P	Berbyelva C (2007–08)	87	0.33	5	139	AD
						Farmed pool (2005–2010)	375	0.37	17	491	FA

Rivers are sorted north to south.

Population = name of river with postscript letter H = historical sample, C = contemporary sample. N = number of fish included in the genetic analyses, uHE = unbiased heterozygosity, HWE = number of deviations from Hardy Weinberg Equilibrium *P <* 0.05 (over the total of 72 loci), LD = number of times that linkage disequilibrium was observed between pairs of loci within any single sample at P < 0.05 (from a total of 72 loci = 2556 pair-wise combinations), Sample type = AD adults captured within river by angling, P parr, SM smolt, FA = taken from farm. Methods used to compute these summary statistics are detailed in the Methods section, while more extensive population genetic summary statistics per population are presented in Additional file 3: Table S3.

Prior to isolation of DNA, all scale samples were first examined for growth patterns in order to exclude any potential farmed salmon that had escaped from a commercial fish farm, using established methods [37]. The habitat and demographic data for the populations included in the present study, including numbers of escapees observed, are presented online (Additional file 1: Table S1).

### Farmed salmon samples

A total of 375 farmed salmon were analysed in this study. These fish were collected in the period 2005–2010 from 49 separate sources. These included 48 marine cages (approx. 8 salmon per cage) located on 35 commercial farms spanning from the south to the far north of Norway, in addition to eight escapees captured in the sea. These samples were picked from approximately 6000 farmed fish that had been previously genotyped with microsatellites in association with a forensic service conducted by the Institute of Marine Research to identify the farm of origin for escaped salmon for the legal authorities [32,38-41]. The aim of this sampling strategy was to generate a pool of farmed fish representing the genetic diversity of farmed fish in Norway.

### Genotyping

DNA was extracted in 96-well format using Qiagen DNeasy blood and tissue kit. Each plate contained two or more negative control wells. DNA aliquots of these samples were sent to the Centre for Integrative Genetics (CIGENE) in Norway for SNP (n = 99) analysis using a Sequenom platform. A list of the markers, their NCBI assay details and linkage map positions are available (Additional file 2: Table S2).

Seventy of the SNP markers genotyped here were selected from the panel of SNPs that have been suggested to be collectively diagnostic for farmed and wild Norwegian salmon [31]. These 70 SNPs include the ten top ranking loci, 54 of the 60 top ranking loci, and a further 16 selected from the top 200 ranking loci (these were selected in order to create working Sequenom assays Additional file 2: Table S2). As the collectively diagnostic loci identified by Karlsson et al. (2011) were ranked based upon their F_ST_ between a pool of farmed and pool of wild Atlantic salmon, and the sequential difference in F_ST_ between each locus was very small, it is not expected there is any specific combination of loci required to create the genetic signal permitting identification of farmed and wild salmon. However, the ability of the collectively diagnostic markers used in the present study to differentiate farmed and wild salmon has been empirically evaluated here (see Results). It is furthermore important to note that seven of the wild populations used in the marker identification study conducted by Karlsson et al. (2011) overlap with populations in the present study (Alta, Namsen, Surna, Lærdalselva, Vosso, Figgjo, Numedalslågen). While this can potentially cause ascertainment bias, this has been considered when interpreting results. In addition to the 70 diagnostic SNPs, a further 29 SNPs were also genotyped. These were selected as putatively neutral SNPs that are known to be polymorphic in Norwegian salmon.

### Statistical data analysis

In order to investigate temporal genetic stability in the 20 populations, and the direction of any potential changes, the data set was organized into the historical and contemporary samples. The pool of farmed salmon was only used for specific tests and the simulations to quantify gene flow (see below). For all computations, the data sets were divided into the loci that are collectively diagnostic between farmed and wild Atlantic salmon and the randomly selected loci.

Genotypic data was first organized in the program MSA, coding the nucleotides A, C, G and T as alleles 1–4 [42]. MSA was used to compute F_ST_ values (global and pair-wise) and compute significance levels associated with these tests using the Fisher’s exact method as implemented in the program. F_ST_ values were all computed using the Weir and Cockerham estimator [43]. Confidence intervals associated with the global F_ST_ values for the historical and contemporary sets of samples were computed from the distribution of 1000 F_ST_ values calculated from 1000 bootstraps where 35% of the individuals from each population were randomly re-sampled. This latter test was computed in the program R (R development team).

Population-specific summary statistics, i.e., numbers of alleles, heterozygosities, numbers of deviations from Hardy Weinberg Equilibrium (HWE), and the numbers of times that linkage disequilibrium (LD) was observed between pairs of loci within each sample were computed in the program GENALEX v6. [44] using program default parameters for these tests. Where appropriate, statistical significance levels were tested against *P* < 0.05, *P* < 0.001 and Bonferroni corrected threshold levels.

GENALEX v6. was also used to create principal component analysis plots (PCA) based upon a matrix of F_ST_ values. This was conducted using the program’s default values in order to investigate spatio-temporal population genetic structure in addition to the direction of any observed temporal changes in relation to the pool of farmed fish. Isolation by Distance (IBD) analyses on the historical and contemporary set of samples was conducted using the Mantel test as implemented in the R package “Vegan”. The test was computed with input data from a matrix of pair-wise F_ST_ values, and, the pair-wise distances between river mouths in kilometers.

Genetic assignment tests, using direct assignment and exclusion and different combinations of samples and loci sets, were conducted in the program GENECLASS2 [45], using a specific algorithm for the computations [46] and probabilities of *P* < 0.05 and *P* < 0.001 for exclusion. Bayesian clustering analysis was computed in the program STRUCTURE [47,48]. This was used to look at temporal genetic changes within each wild population one at a time by including the historical and contemporary sample. Analysis parameters included an admixture model, correlated allele frequencies, and assuming no population prior. Each analysis with this program consisted of 5 replicate runs for K = 1-5, each with a burn-in of 250 000 replications, and a run length of 500 000 Markov chain Monte Carlo (MCMC) iterations.

In order to investigate the statistical power of the different sets of genetic markers implemented here, several tests were computed. First, the distribution of pair-wise F_ST_ between two groups of 100 randomly selected fish from within each of the groups (wild and farmed) was plotted following 1000 bootstraps. This was conducted for farmed vs. wild, wild vs. wild and farmed vs. farmed using the diagnostic markers and the randomly selected markers. Next, the assignment power of these sets of loci was examined in STRUCTURE. Due to the fact that there were more diagnostic loci than randomly selected loci, and that assignment power is influenced by the number of loci, a sub-set of the diagnostic loci (equal to the number of randomly selected loci) was also used to compute these assignment tests. The following routine was repeated 1000 times for each set of SNPs: 100 farmed fish were sampled as the learning farmed sample, 100 wild fish among all populations were sampled as the learning wild samples, then one fish was sampled randomly to constitute the unknown individual to identify. STRUCTURE was then run with 50 000 burn-in and 500 000 iterations, at K = 2, using population information and population flag, so that population information is only used for the learning samples, but not for the unknown individual. The accuracy of assignment of this individual to either the farmed or the wild salmon groups was compared for the different sets of SNPs using the threshold probability of 0.6. The re-sampling simulations were performed in R, and the STRUCTURE runs with ParallelStructure R package (https://r-forge.r-project.org/projects/parallstructure/) [49].

### Simulations to quantify gene flow

Two alternative methods were employed to quantify the amount of gene flow that would be required from an alien population to cause the observed temporal genetic change within each wild population, computed as F_ST_ between each wild population’s historical and contemporary sample. The simulations were coded and executed in R using previously published scripts to simulate realistic genetic introgression using gametes sampled randomly from the donating and recipient populations [28]. Average generation time for each wild population was set to five years, thus, the number of generations used to simulate gene flow was set as the number of years between the historical and contemporary sample for each population, divided by five. An effective population size (*N*_*E*_) of 200 for all populations except those displaying an *N*_*E*_ less than this in which case the observed *N*_*E*_, as reported previously [21], using a one sample linkage disequilibrium based method implemented in LDNE 1.31 software [50], was used.

First, the posterior point estimate of migration rate (M) was inferred by an ABC algorithm [33]. The routine to quantify this gene flow consisted of the following steps: 1. Determine a prior distribution for migration rate *M* (e.g., M ~ N(0.1,0.1)). 2. Simulate *n* scenario of introgression where the value of *M* is sampled from the prior distribution, and compute the F_ST_ between historical and each simulated population. 3. Calculate the vector *s* of the *n* differences between simulated and observed F_ST_: s: (s_*1*_, s_*2*_,… s_*n*_) where s_i_ = (F_STO_- F_ST*i*_). 4. Solve the linear regression: M= α+βs+ϵ (Where ϵ is a vector of residuals, s is the vector estimated in step 3, and α is a constant). The estimate of α gives the expected value of *M* when F_STO_- F_ST*i*_ = 0. 5. Update the prior distribution of M with estimated distribution of α. 6. Repeat step 2–5 until α converges to a stable estimate.

To account for the standard deviation of the observed F_ST_ between historical and contemporary sample in the estimation of M, the posterior distribution of M was also estimated with an alternative “fixed migration rate” approach that consisted of the following steps: 1. Simulating genetic introgression from alien population with a fixed migration rate *M* per generation. Tested values of M were from 1 to 80%, every 1% between 1 and 20, and every 5% between 20 and 80%. 2. From the set of simulated populations (each corresponding to a value of *M* = 1, 2, 3, 4…80% migration), selecting 1000 among those that gave an F_ST_ that fit within the 95% confidence interval of the observed F_ST_. 3. Mean and standard deviation of migration rate from the 1000 selected scenario provide a posterior distribution of (*M*).

For both ABC and fixed migration rate approach, two alternative scenarios were tested. First, the alien population considered for the simulations was a pool of farmed salmon, and second, to account for possible straying, the alien population was the historical population of the geographically nearest neighbour river. The R package ADEgenet [51] was used to generate PCA plots of historical, contemporary and simulated populations from each scenario based on individual genotypes.

## Results

### Data quality

Loci that displayed technically unreliable genotype clustering, or a genotyping coverage <90% in the entire data set, were excluded prior to all statistical analyses. This stringent quality control reduced the total number of loci from 99 to 72. Among the remaining loci were 47 SNPs selected from the collectively diagnostic panel for farmed and wild salmon (including the top ten ranked loci, and 35 of the 60 highest ranking loci) [31], and a set of 25 random SNPs. These panels are hereafter referred to as 47d (diagnostic) and 25r (random). Individual fish displaying genotyping coverage <75% over the remaining 72 loci were also removed from the data set (301 fish removed from 2912 samples). Thus, the final data set for analysis consisted of 2611 individuals genotyped for 72 loci. Within this, a total of 364 178 alleles were successfully scored, giving an overall genotyping coverage of 97%.

### Within population summary statistics

A range of population genetics parameters are summarized for all wild populations and the pool of farmed salmon (Table 1; Additional file 3: Table S3). The unbiased expected heterozygosity (UH_E_) over all 72 loci was very even among wild populations, ranging from a low of 0.33 for both the historical and contemporary samples for Berbyelva, to a high of 0.38 for the historical sample representing the river Ørstadelva. None of the populations displayed clear increases or decreases in this parameter between their historical and contemporary samples.

The parameters HWE and LD have the ability to indicate disturbances in populations due to introgression of genetically distinct fish. At the significance level *P <* 0.05, no wild salmon sample displayed more than five deviations from HWE across the 72 loci. This is the number of observations that are more or less expected by chance at this significance level. From a total of 2556 comparisons within each sample (72 loci pair-wise) at *P <* 0.05, LD was observed from a low of 70 times (2.6%) in Bondalselva contemporary sample, to a high of 243 (9.2%) in the historical Vestre Jakobselva sample. The pool of farmed salmon displayed similar summary statistic values to the wild populations, although increased frequency of HWE and LD was observed in this sample due to it being a mixture of fish from multiple sources (Table 1; Additional file 3: Table S3).

The effective population size for each wild population is presented (Additional file 4: Table S4). Most of the samples and populations displayed *N*_*E*_ 100-1000+. Notable examples of low *N*_*E*_ were Vestre Jakobselva (98), Bondalselva (25), Ørstaelva (94), and Opo (57) for the historical samples, and Vestre Jakobselva (75) and Berbyelva (67) for the contemporary samples. The upper and lower 95% confidence intervals surrounding all estimates were large however, with the upper boundary often reaching infinity.

### Comparison between 47d and 25r

In order to test the statistical characteristics of the putatively diagnostic loci 47d vs. the randomly selected loci 25r, several comparisons were conducted. The number of loci displaying non-overlapping allele frequencies between the pool of farmed salmon and all 20 historical wild salmon samples separately was 15 for 47d (32%) and 3 for 25r (12%) (Additional file 3: Table S3). Thus, while 32% of the loci from 47d displayed non-overlapping allele frequencies, the majority did not. Of these non-overlapping loci, some displayed moderately strong differentiating frequencies but not all. Based upon the distribution of the F_ST_ values between 100 farmed and 100 wild salmon (historical samples) randomly re-sampled from the data set 1000 times, 47d displayed approximately double F_ST_ than 25r (Additional file 5: Figure S1).

Genetic assignment values as computed in STRUCTURE revealed that assignment to correct source (farmed pool or wild pool) was higher for 47d than 25 randomly re-sampled loci from 47d (“25resam”), and from 25r (Correctly assigned wild: 69, 63, and 23%; correctly assigned farmed: 82, 58, and 30% when using 47d, “25resam” and 25r respectively). Together, these tests demonstrate that the diagnostic loci contain far greater statistical power to differentiate farmed and wild salmon than the randomly selected loci.

### Temporal F_ST_ changes

Over the full set of 72 loci, the number of populations displaying significant temporal genetic changes at *P* < 0.05 and *P* < 0.001 was 11 and five respectively. For either 47d or 25r at *P* < 0.05 (Table 2), the number of populations displaying temporal changes was 12. At the more stringent threshold *P <* 0.001, this number dropped to five. Looking specifically at 47d, the three rivers displaying the largest temporal genetic changes were all located on the west of Norway; Opo, Vosso and Loneelva respectively. In all three of these rivers, temporal genetic changes, and measured by F_ST_, were greater for 47d than 25r, a trend observed in a total of 13 of the 20 populations in the entire data set.

**Table 2:** Temporal genetic stability in the 20 rivers ordered north to south

**Population**	**Pair-wise F**_**ST **_**historical vs Contemporary**				**Exclusion of contemporary sample from historical *P*** **< 0.001**			
	**72**	**47d**	**25r**	**22**	**72**	**47d**	**25r**	**22**
	**SNPs**	**SNPs**	**SNPs**	**Micros**	**SNPs**	**SNPs**	**SNPs**	**Micros**
Neiden	−0.0002	−0.0017	0.0025	0.0009	0%	0%	0%	6%
V. Jakobselv	0.0054**	0.0067**	0.0029	0.0064**	7%	5%	1%	16%
Alta	0.0040*	0.0059*	0.0003	−0.0002	2%	3%	0%	2%
Reisa	0.0023	0.0036	−0.0004	0.0041*	6%	4%	0%	15%
Målselv	0.0038	0.0082*	−0.0043	−0.0026	7%	10%	0%	13%
Roksdalsvass.	0.0037*	0.0066*	−0.0016	0.0014	1%	1%	0%	20%
Namsen	0.0042**	0.0027*	0.0068*	0.0013*	1%	2%	0%	9%
Surna	0.0093	0.0010	−0.0055	0.0025	2%	2%	0%	12%
Eira	−0.0039	−0.0051	−0.0016	0.0005	5%	0%	0%	34%
Bondalselva	0.0034	0.0041	0.0022	0.0043	8%	8%	8%	14%
Ørstaelva	0.0006	0.0005	0.0008	0.0003	6%	3%	0%	6%
GaulaSF	0.0011	0.0025	−0.0015	0.0001	0%	2%	1%	0%
Lærdalselva	0.0005	0.0001	0.0011	0.0015	2%	4%	0%	17%
Vosso	0.0125**	0.0168**	0.0049	0.0070**	12%	16%	2%	58%
Loneelva	0.0071**	0.0105**	0.0003	0.012**	4%	2%	0%	52%
Opo	0.0200**	0.0216**	0.0172**	0.0258**	8%	2%	8%	100%
Etne	0.0038*	0.0062**	−0.0007	0.0006	1%	0%	1%	5%
Figgjo	0.0050*	0.0040*	0.0071*	0.0048**	3%	0%	0%	38%
Numedalslågen	0.0036*	0.0023	0.0058*	0.0032*	4%	0%	0%	29%
Berbyelva	0.0032*	0.0042*	0.0015	0.0053**	1%	3%	0%	16%

Stability is quantified by pair-wise F_ST_ and percent exclusion of the contemporary sample from the historical sample. Both parameters measured using sub-sets of SNPs and previously published data using microsatellites.

Micros = microsatellite data taken from [21]. Note, the contemporary Vosso sample is not the same as the contemporary Vosso sample used in Glover et al., 2012b. * = significant *P <* 0.05, ** = significant *P <* 0.001.

A statistically significant correlation was observed between the degree of within-river temporal genetic change (as revealed by F_ST_) using 47d and 25r (R^2^ = 0.36 *P* = 0.0049) (Additional file 6: Figure S2). However, the degree of within-river change was more strongly related between 47d and previously published results for these populations based upon 22 microsatellites [21] (R^2^ = 0.63 *P* < 0.0001) (Additional file 6: Figure S2).

Genetic assignment tests were used to investigate the probability of excluding the composite genotype for each individual fish taken from the contemporary sample from the historical genetic profile of the population. This compliments temporal F_ST_ analyses as it also reflects distribution of genotypes among individual fish in the contemporary sample. At *P <* 0.001, the percentage of fish from the contemporary sample that could be excluded from the historical sample ranged from a low of 0% for the river Neiden, to a high of 12% for the river Vosso. Using 47d or 25r only, the percentage of fish from the contemporary sample that could be excluded from the historical profile were generally lower than with the 72 loci (Table 2). In comparison with previous data on these populations using 22 microsatellites [21], the exclusion levels achieved with even the full set of 72 SNPs were strikingly lower (Table 2).

Bayesian clustering analysis for each population separately revealed clear temporal genetic changes in the rivers Opo and Vosso when computed using data from all 72 loci (Additional file 7: Table S5). The results of admixture analysis for the remaining rivers, using either all 72 loci or 47d was either very subtle, or non-existent (Additional file 7: Table S5).

### Comparisons of wild salmon to the farmed salmon

**Figure 2:**
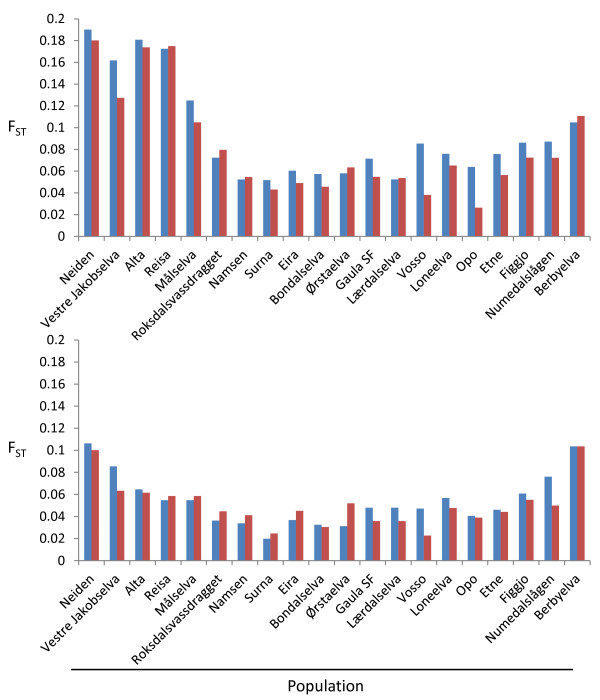
**Pair-wise F**_**ST **_**between the historic sample for each wild population and the farm sample (blue–left bar), and the contemporary sample for each wild population and the farm sample (red–right bar), computed using 47 diagnostic SNPs (top), and 25 randomly selected SNPs (bottom).** Populations are ordered north to south.

Computed pair-wise, all historical and contemporary samples representing the wild populations displayed statistically significant differences to the pool of farmed salmon, using both sets of SNPs separately, and following adjustment for multiple testing (all *P* < 0.001). Several trends can be extrapolated from these comparisons (Figure 2). First, all F_ST_ values computed pair-wise between each historical wild sample and the farmed sample was higher for 47d compared to 25r (Anova DF _1:38_, F = 12.1, *P* < 0.001). This is consistent with the results of the section above dedicated to comparing the statistical properties of 47d and 25r. Second, a geographic trend to the pair-wise difference between each wild population’s historical sample and the pooled farmed sample was present. Populations from the far north and south of Norway displayed much greater pair-wise F_ST_ estimates to the farmed sample than populations from mid- and western Norway displayed to the farmed sample. This was detected in 47d and 25r, but more pronounced in the former. These observations suggest that a population in northern Norway is likely to display a greater genetic change compared to a population from mid Norway for the same level of farmed salmon introgression.

**Figure 3:**
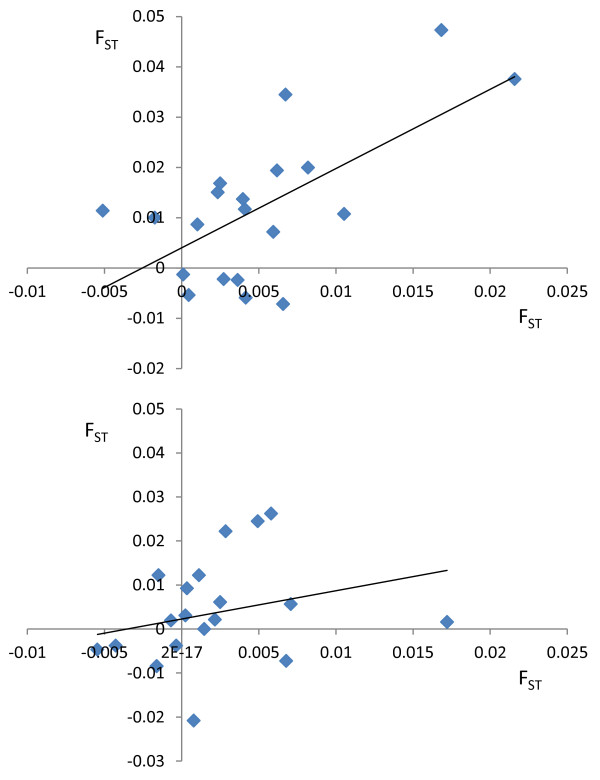
**Relationship between the temporal genetic change observed within any given population (i.e., pair-wise F**_**ST **_**between the historical and ceontemporay sample) on the X axis, and the difference in pair-wise F**_**ST **_**between each populations historical sample and the farm sample, and that same populations contemporary sample and the farm sample, placed on the Y axis.** These relationships are computed with 47 diagnostic SNPs (top) (R^2^ = 0.41 *P* = 0.0022) and 25 randomly selected SNPs (bottom) (R^2^ = 0.07 *P* = 0.25). Each point represents the above mentioned relationship for each of the 20 populations.

Looking specifically at 47d, the pair-wise difference between the wild sample and the pool of farmed salmon decreased with time in 14 of the 20 populations. This was most noticeable for Vosso, Opo and Vestre Jakobselva (Figure 2). For example, the pair-wise F_ST_ between Vosso and the farmed sample dropped from 0.085 to 0.038. Furthermore, a significant positive relationship between temporal genetic change within each river, and the change in pair-wise F_ST_ between that specific rivers historical sample and the farmed salmon sample, and that rivers contemporary sample and the farmed sample, was observed for 47d (R^2^ = 0.41, *P* = 0.0022), but not for 25r (R^2^ = 0.07, *P* = 0.25) (Figure 3). Thus, analyses based upon 47d demonstrate that where rivers display temporal genetic change, it is largely in the direction of the farm sample.

Assignment tests were also used to exclude the 375 genotyped farmed fish from each wild population (Additional file 8: Figure S3). For both the historical and contemporary samples, the exclusion percentages followed a similar geographic pattern as observed for the pair-wise F_ST_ between each population and the farmed sample (Figure 2). The percentage of farmed fish that could be excluded from each wild fish sample decreased between the full set of 72 loci to 47d and 25r. The relative change for any given population, i.e., the drop in exclusion of the farmed fish between the historical sample and contemporary sample (which is more interesting in this specific context than the absolute level of exclusion) was still very noticeable for 47d.

**Figure 4:**
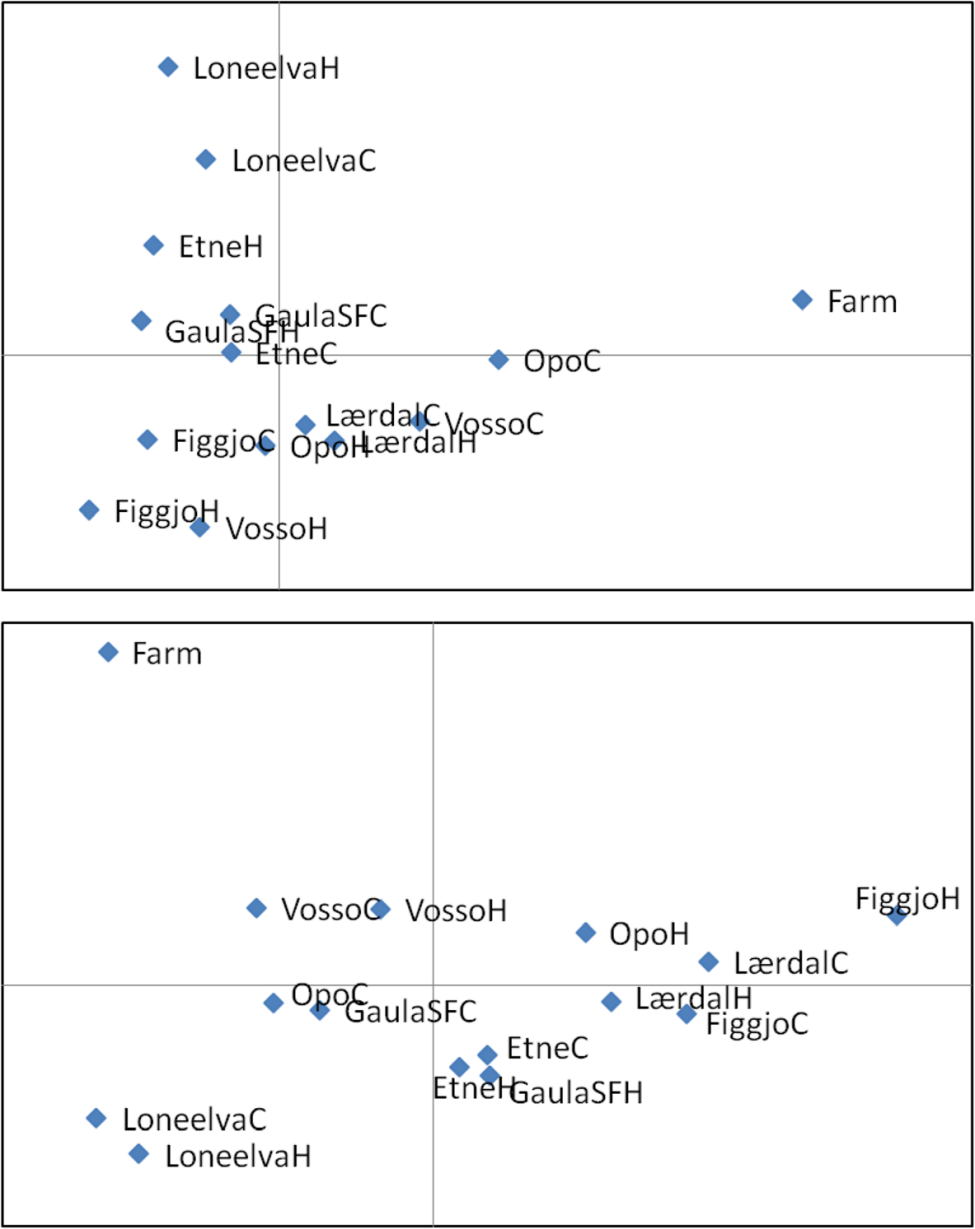
**Principal compont analysis for the historical (H) and contemporary (C) samples for the seven rivers grouped into the west of Norway.** This region includes the three rivers displaying the greatest temporal genetic changes in the entire study. Plots are based upon 47 diagnostic SNPs (top) and 25 randomly selected SNPs (bottom). X-and Y axes explain 35, 23%, and 29, 23% for the top and bottom figures respectively. PCA plots for all 20 rivers arranged into four geograpahic regions are also presented online (Additional file 9: Figure S4).

An alternative way of investigating the direction of the observed temporal genetic changes in the wild populations is to conduct PCA analysis. A PCA plot for the seven populations located in the west of Norway, which includes the three rivers displaying the highest temporal genetic changes in the complete data set for 47d, revealed that six of the seven populations displayed genetic changes in the direction of the farm sample (Figure 4). Notably, none of them displayed temporal genetic changes away from the farm sample. While some other populations located in the other regions of Norway displayed temporal genetic changes in the approximate direction of the farm sample, this was not observed for all samples displaying temporal genetic changes (Additional file 9: Figure S4).

### Quantification of gene flow required to cause observed changes

The estimated level of farmed salmon introgression required to cause the observed temporal genetic changes in the 20 populations ranged from 2-47% and 7-41% using the ABC and fixed migration methods respectively (Table 3). For the two populations displaying the greatest temporal genetic change (Opo and Vosso), the estimated level of farmed salmon introgression ranged from 36-41% and 33-47%. In contrast, the simulated level of gene flow required from the nearest neighbor population, i.e., straying, to cause the observed temporal changes in these two populations was much greater, approximately 80-82% and 46-61% for the ABC and fixed migration methods respectively. This is far greater than straying rates typical for this species. For both computation methods, the introgression rate required by the straying scenario was always far greater than introgression required by the farmed escapees scenario. This is likely to reflect the genetic similarity of the nearest neighbor population to each recipient wild population, and how distinct each wild population’s historical sample was to the farmed sample, the latter of which follows a geographic trend as demonstrated earlier (Figure 2).

**Table 3:** Estimated percentage introgression of farmed salmon, or the nearest wild population required to cause the observed temporal genetic changes

**Population**	**Admixture from farm salmon**				**Admixture from nearest neighbor population**			
	**ABC**		**Fixed migr.**		**ABC**		**Fixed migr.**	
	**Pr. Gen.**	**Total**	**Pr. Gen.**	**Total**	**Pr. Gen.**	**Total**	**Pr. Gen.**	**Total**
Neiden	0.004	0.022	0.014	0.073	0.029	0.139	0.028	0.142
	±0.009	±0.051	±0.006	±0.031	±0.035	±0.152	±0.023	±0.110
V. Jakobs.	0.031	0.116	0.032	0.116	0.130	0.385	0.175	0.414
	±0.005	±0.030	±0.020	±0.063	±0.011	±0.045	±0.133	±0.223
Alta	0.031	0.116	0.043	0.117	0.114	0.350	0.136	0.359
	±0.004	±0.024	±0.031	±0.062	± 0.013	±0.042	±0.092	±0.198
Reisa	0.017	0.066	0.035	0.126	0.040	0.143	0.116	0.319
	±0.006	±0.028	±0.023	±0.070	±0.017	±0.061	±0.084	±0.190
Målselv	0.054	0.190	0.051	0.167	0.083	0.273	0.097	0.280
	±0.006	±0.032	±0.041	±0.114	±0.004	±0.030	±0.071	±0.175
Roksdalsvass	0.055	0.192 ±	0.063	0.199	0.071	0.239	0.077	0.220
	±0.004	0027	±0.054	±0.146	±0.004	±0.029	±0.052	±0.143
Namsen	0.01	0.062	0.033	0.170	0.056	0.252	0.027	0.126
	±0.013	±0.074	±0.020	±0.090	±0.047	±0.210	±0.037	±0.162
Surna	0.010	0.038	0.083	0.243 ±	0.039	0.137	0.255	0.507
	±0.014	±0.057	±0.073	0179	±0.046	±0.138	±0.188	±0.253
Eira	0.016	0.053	0.042	0.145	0.033	0.116	0.092	0.268
	±0.031	±0.100	±0.032	±0.097	±0.035	±0.141	±0.069	±0.168
Bondalselva	0.026	0.098	0.092	0.263 ±	0.122	0.363	0.301	0.547
	±0.015	±0.055	±0.081	0193	±0.029	±0.074	±0.223	±0.270
Ørstaelva	0.014	0.050	0.070	0.217	0.050	0.165	0.304	0.554
	±0.019	±0.068	±0.060	±0.154	±0.042	±0.129	±0.219	±0.273
GaulaSF	0.022	0.085	0.050	0.165 ±	0.028	0.105	0.097	0.280
	±0.009	±0.038	±0.041	0115	±0.008	±0.034	±0.070	±0.172
Lærdalselva	0.015	0.088	0.027	0.169	0.019	0.115	0.026	0.154
	±0.027	±0.142	±0.013	±0.077	±0.028	±0.144	±0.021	±0.112
Vosso	0.077	0.360	0.102	0.410	0.107	0.459	0.283	0.605
	±0.003	±0.032	±0.015	±0.200	±0.005	±0.033	±0.140	±0.145
Loneelva	0.094	0.307	0.075	0.226	0.124	0.375	0.109	0.311
	±0.010	±0.029	±0.069	±0.166	±0.006	±0.031	±0.071	±0.172
Opo	0.084	0.474	0.061	0.331	0.238	0.817	0.338	0.804
	±0.004	±0.044	±0.053	±0.209	±0.020	±0.029	±0.205	±0.197
Etne	0.044	0.197	0.040	0.170	0.069	0.274	0.098	0.337
	±0.005	±0.033	±0.030	±0.107	±0.033	±0.117	±0.067	±0.189
Figgjo	0.009	0.060	0.029	0.178	0.018	0.120	0.044	0.236
	±0.010	±0.069	±0.013	±0.077	±0.023	±0.125	±0.035	±0.162
Numedals.	0.007	0.030	0.040	0.143	0.021	0.078	0.060	0.191
	±0.006	±0.026	±0.030	±0.089	±0.010	±0.041	±0.046	±0.130
Berbyelva	0.025	0.093	0.040	0.138	0.045	0.163	0.070	0.219
	±0.012	±0.049	±0.029	±0.089	±0.005	±0.030	±0.050	±0.138

Estimations of introgression are based upon approximate Bayesian computation (ABC), or a fixed migration rate based simulation (Fixed migr.). Populations ordered north to south.

ABC = estimation of introgression using approximate Bayesian computation, Fixed migr. = estimation of introgression using a fixed migration rate estimator (see Methods).

**Figure 5:**
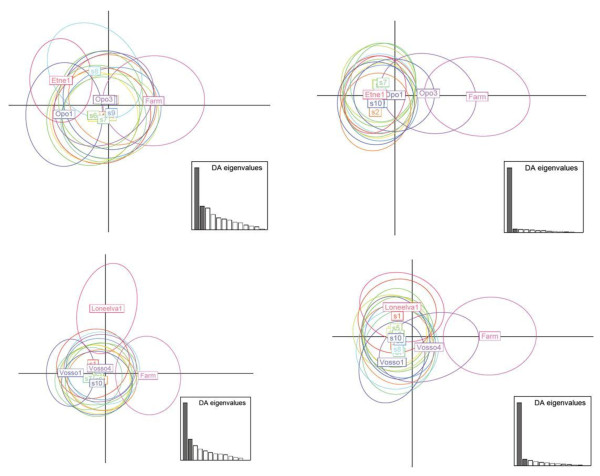
**Principal component analysis depicting observed historical and contemporary samples for the river Opo (top panels), and Vosso (bottom panels), including the farmed sample and nearest wild population, following simulated introgression from the farmed sample (left panels), and simulated introgression from the nearest neighbor (right panels).** In each case, the results of ten independent simulations, S1-S10 are presented. The text box represents the center point of the observations with the 95% confidence interval represented by the ellipse.

The observed temporal genetic changes for the rivers Opo and Vosso, in relation to the simulated genetic change by introgression of farmed fish, or the nearest neighboring population, confirmed results presented earlier that the genetic changes observed in these two populations were clearly directional to the farmed fish, and not likely to be cause by natural straying (Figure 5). Put simply, the observed temporal genetic change in both of these populations overlapped almost perfectly with the simulated genetic change caused by the pool of farmed salmon, but not by the nearest neighbor (Figure 5). For other populations that displayed more modest temporal genetic changes however, this pattern was more difficult to elucidate from the simulation-based PCA plots due to the fact that the changes were small and distributions overlapped.

### Observed frequency of escapees and genetic changes

**Figure 6:**
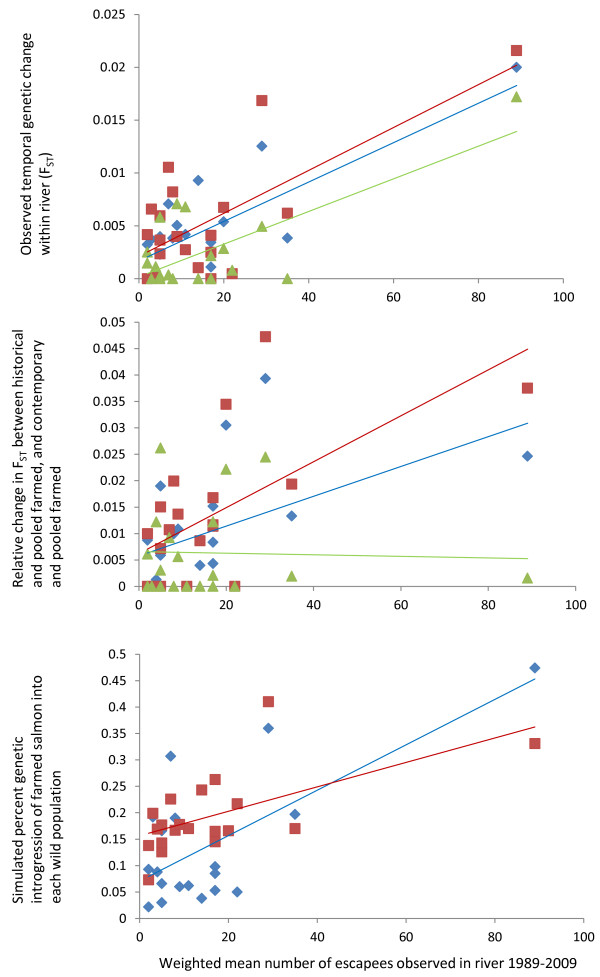
**Relationship between the weighted mean number of escapees observed in each of the rivers in the period 1989–2009 (X axis), and the following three sets of estimators of genetic change in the 20 populations (Y axes).** Top graph Y axis = the observed within-river temporal genetic change as measured by pair-wise F_ST_ using all 72 loci (blue diamonds), 47d (red squares) and 25r (green triangles). Middle graph Y axis = the relative change (%) in pair-wise F_ST_ between a population’s historical sample and the pooled sample of farmed salmon, and the same populations contemporary sample and the pooled sample of farmed salmon using all 72 loci (blue diamonds), 47d (red squares) and 25r (green triangles). Bottom graph Y axis = the simulated level of farmed salmon genetic introgression required in each population to cause the observed temporal genetic changes as based upon the ABC method (blue triangles) and the fixed migration method (red squares), both of these simulations computed using 47d. Significance levels and R^2^ values for all relationships are provided in Table 4.

A set of correlations between the frequency of farmed escapees observed on the spawning grounds in the time-period in which the present study is conducted, and various genetic parameters produced with the present study for these populations are summarized (Table 4), and plotted graphically (Figure 6). All genetic parameters gave stronger correlations with the weighted mean frequency of escaped salmon than the un-weighted mean frequency of farmed escaped salmon. This suggests that the weighted estimate more accurately reflects the true numbers of escapees in the populations as it corrects for noise in the estimations (i.e., small sample sizes in some years etc.).

**Table 4:** Relationships between the frequency of farmed escaped salmon in the spawning population, and observed changes in various genetic parameters

**Statistic**	**Un-weighted mean**	**Weighted mean**
Pair-wise F_ST_ 72	0.19 (0.052)	0.52 (0.0003)
Pair-wise F_ST_ 47d	0.17 (0.067)	0.44 (0.0014)
Pair-wise F_ST_ 25r	0.06 (0.30)	0.41 (0.0022)
F_ST_ to farm 72	0.13 (0.11)	0.22 (0.039)
F_ST_ to farm 47d	0.25 (0.024)	0.37 (0.0045)
F_ST_ to farm 25r	<0.01 (0.72)	<0.01 (0.99)
ABC introgression	0.16 (0.08)	0.47 (0.0007)
Fixed migration rate introgression	0.25 (0.025	0.36 (0.005)

Values computed are R^2^ (*P*-value). Number behind statistics refers to number of SNP loci used, introgession computations only compared for 47d.

Pair-wise F_ST_ = observed value between historical and contemporary sample for each river; F_ST_ to farm = absolute difference in pair-wise F_ST_ between the populations historical sample and the farm sample, and between the populations contemporary sample and the farm sample, ABC introgression = estimated introgression of farmed salmon (Table 3) using ABC method, Fixed migration rate introgression = estimated introgression of farmed salmon (Table 3) using the fixed migration rate method. Un-weighted mean = mean% of farmed salmon observed on the spawning grounds for these rivers in the period 1989–2009 [5,52], weighted mean = weighted average percentage of farmed salmon in the population combining data from both sports-fishing and spawning population samples [53].

### Spatio-temporal variation

The pair-wise F_ST_ values among all samples included in this study, including associated *P* values, are presented online (Additional file 10: Table S6). Using all sets of markers, global F_ST_ estimates among the 20 wild populations decreased significantly with time (Table 5). Nevertheless, a geographic pattern to the genetic structure was still evident with both 47d and 25r (Additional file 11: Figure S5), and there was no detectable change in IBD (Table 5). This means that while overall variation among the wild samples decreased with time, this happened “evenly” which did not influence the relationship between genetic and physical distance.

**Table 5:** **Spatio-temporal analysis of population genetic structure (global F**_**ST**_**) including isolation by distance (IBD)**

**Loci**	**Historical samples**				**Contemporary samples**				**Global F**_**ST **_**change?**
	**IBD**	***P***	**Global F**_**ST **_**(SD)**	***P***	**IBD**	***P***	**Global F**_**ST **_**(SD)**	***P***	***P***
72	0.79	<0.001	0.055 (0.004)	<0.001	0.77	<0.001	0.046 (0.003)	<0.001	≤0.003
47d	0.79	<0.001	0.061 (0.004)	<0.001	0.76	<0.001	0.051 (0.004)	<0.001	≤0.001
25r	0.60	<0.001	0.043 (0.006)	<0.001	0.66	<0.001	0.036 (0.004)	<0.001	0.046

IBD = isolation by distance with associated *P* value, Global F_ST_ = global F_ST_ among the wild populations with standard error in brackets and associated *P* value. Hist. Vs. Cont. = statistical significance whether or not the change in global F_ST_ with time was significant or not.

## Discussion

Norway is the world’s largest producer of farmed Atlantic salmon, and has over 200 rivers supporting native Atlantic salmon populations. Many Norwegian populations have displayed moderate to high frequencies of domesticated farmed escapees on the spawning grounds for two decades or more [1,4,54]. At the same time, Norwegian farmed salmon originated from native Norwegian populations approximately ten generations ago. Thus, it follows that Norway is not only the country where the potential genetic interaction between farmed escaped salmon and wild conspecifics is the most extensive, it represents the country in which the statistical challenges to quantify genetic introgression of farmed escapees are the most demanding. The present study addressed this situation by genotyping a sub-set of SNP markers (47d) that have been reported to be collectively diagnostic for farmed and wild salmon [31], and by implementing ABC simulations to quantify introgression of farmed salmon.

The most important results of this study can be summarized as follows: 1. All populations displaying significant temporal genetic changes with 47d became more similar to the pooled of farmed salmon. Furthermore, the stronger the temporal genetic change with 47d, the more similar it became to the pool. This strongly suggests that where populations displayed clear temporal genetic changes, introgression of farmed fish has been the primary cause 2. This is the first study to estimate cumulative introgression of farmed salmon in any native Atlantic salmon population. Estimations ranged between 2–47% and 7–41% per population using the ABC and fixed migration simulation methods respectively. It is concluded that while the level of introgression has been population specific, farmed salmon have heavily introgressed in some wild Norwegian populations.

### Are the diagnostic SNPs universally informative

The panel of diagnostic SNPs used here (47d) represents a sub-set of the markers recently identified as collectively diagnostic for farmed and wild Norwegian salmon [31]. The panel 47d included 35 of the top 60 loci ranked by Karlsson et al. (2011), including the top 10 ranking loci, and a further set of 12 loci taken from the ranks 60–200. While this study has not used the exact combination of markers reported to be collectively diagnostic, which is in large part due to poor genotyping quality for many of those markers, 47d still provides similar characteristics of the panel reported to be collectively diagnostic. This is based upon the comparisons reported here showing the greater level of signal for 47d vs. 25r for a variety of statistic parameters (e.g., Figures 2, 3, Additional file 5: Figure S1, Table 2).

Seven of the populations investigated here overlap with some of the populations used to identify the diagnostic SNPs in Karlsson et al. [31]. However, there were no sign that ascertainment bias influenced the results of the present study. This is based upon the following observations: 1. The population displaying the greatest temporal change with 47d was not included in the ascertainment panel, 2. Pair-wise F_ST_ between each wild population and the pool of farmed salmon showed a clear geographic trend (Figure 2), with populations in the north of Norway displaying the greatest difference to the farmed pool. This is likely to reflect a combination of the fact that there is a distinct evolutionary divide between Atlantic salmon populations in the north and rest of Norway [21], and that Norwegian farmed strains were largely sourced from wild populations south of the observed evolutionary divide [29,55]. Thus, it follows that detection of introgression in native populations in Northern Norway should be easier to detect with this set of markers than in populations for example from mid- and western Norway. Consequently, the present study serves to validate the usefulness of the collectively diagnostic markers in populations other than those in which the marker identification was conducted. Given that Atlantic salmon populations display highly significant population genetic structuring throughout the Atlantic [6,7], it is likely that these markers will also serve useful to identify introgression of Norwegian farmed salmon in native populations outside Norway.

Assignment tests using 47d provided less statistical power to reject individual salmon from the historical baseline than with microsatellites for all 20 populations (Table 2). This is probably due to the fact that assignment power is strongly influenced by total number of alleles in the data set [39,56], and the fact that a microsatellite data set based upon 22 markers [21] has approximately 3–4 times more alleles than 47 SNPs. Inclusion of more of the collectively diagnostic SNPs identified by Karlsson et al. (2011) would increase these assignment statistics. However, in order to accurately identify the ancestry of hybrids beyond the second admixed generation, it has been suggested that 50 or more ancestry diagnostic markers (i.e., fixed allele differences) are required [57,58]. Only 15 markers from the panel 47d displayed non-overlapping allele frequencies between the pool of farm salmon and all historical samples for each wild population. Furthermore, the allele frequency differences for these non-overlapping markers were not close to fixation between groups (Additional file 3: Table S3). Thus, while the markers identified by Karlsson et al. (2011) provide more information to differentiate farmed and wild salmon than randomly selected markers, the identified markers are more correctly regarded as “collectively informative” than diagnostic. Clearly, there is a need to identify more informative genetic markers on the domesticated/wild interface. As farmed salmon outgrow wild salmon approximately 2–3 times under hatchery conditions [59,60], it is suggested that there is significant potential to identify markers tightly linked with this trait that has been selected for in all farmed strains [29,61].

### ABC and fixed migration estimations of introgression

Admixture between hatchery fish released deliberately into the wild and native populations has been computed in other species and systems, for example brown trout (*Salmo trutta*) in Danish rivers [25]. Often, admixture has been estimated using Bayesian clustering implemented for example in the program STRUCTURE [47,48]. Here, clustering analysis was able to reveal temporal changes in the populations Opo and Vosso (the two populations displaying the largest temporal changes), however, in other rivers, also those displaying statistically temporal genetic changes, this analysis did not reveal changes. This is consistent with previous results using microsatellites [21] and is likely to be caused in part by the fact that the observed genetic changes for most of the rivers were low to modest, and therefore under the detection potential for STRUCTURE.

Introgression of farmed salmon was estimated here using the simulation approach. As it is implemented, the ABC routine finds the point estimate of migration rate *M* that best explains the observed F_ST_ between historical and contemporary population. However this approach does not take into account the possibly large confidence intervals around the observed F_ST_. In many of the populations studied here, the genetic distance between historical and contemporary samples was not significantly different from zero. Therefore an alternative approach was also employed to better account for the uncertainty around the observed F_ST_. By choosing a set of scenarios where the genetic distance between historical and simulated population fitted within the 95% confidence interval of the observed F_ST_, the second approach reflects the range of variation around the posterior mean of *M*, accounting for the uncertainty around the observed F_ST_. The standard deviation of *M* given by ABC estimation only accounts for the variation induced by random gamete sampling of our simulations and is thus lower than the standard deviation of *M* given by fix migration rate approach. This last approach explores the possible values of *M* stepwise with predefined steps (1,2…20%) and is deemed to be less accurate to estimate the posterior mean of *M* than the ABC routine that converges gradually to an optimum value. We therefore present the results from the two methods as complementary. Other alternative approaches could also have provided reliable estimates of posterior distribution of *M*, such as rejection based Bayesian Inference [33,62], that would have estimated both posterior mean and standard deviation of *M*, taking into account the uncertainty around the observed F_ST_. However, algorithms that estimate the value of a parameter based on its posterior likelihood are sensitive to the shape of the likelihood curve or “likelihood landscape”; a leptocurtic curve would result the algorithm to converging rapidly to the optimum value of the estimated parameter, whereas a platicurtic curve would result in the algorithm converging slowly. In the present data, F_ST_ between historical and contemporary samples were close to zero with large standard deviations for some populations. Such combination of small mean value and large standard deviation represents a flat likelihood landscape where the algorithm is slow to converge, and often lacks precision.

### Challenges and alternative approaches to quantify introgression

Some of the challenges to quantify introgression of farmed salmon in native populations have been described in detail in the introduction. Briefly, these include the complicated logistics associated with the distribution of genetic material within the aquaculture industry [38], the fact that there are multiple farmed strains which have and continue to change significantly over time due to splitting and mixing, the fact that over time, gene-flow arises from multiple farmed sources which partially conceals the degree of genetic change in wild populations when studying non-diagnostic markers [28], and finally, the fact that most loci display overlapping allele frequencies between groups of farmed and wild salmon. It is likely that many of these challenges will exist in other countries where farmed and wild salmon coexist, and, in other aquaculture species such as marine fish where interactions between escapees and wild conspecifics have already been registered [63-65].

**Figure 7:**
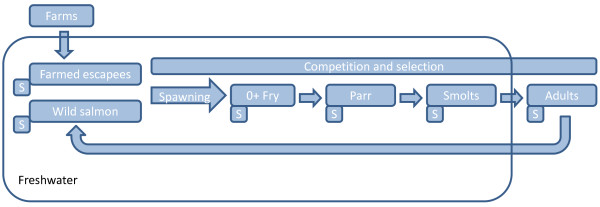
**Illustration of a single-cohort design to quantify introgression of farmed escaped salmon in a wild population, and estimate selection at the different life-history stages.** S = sampling for genetic analysis.

In order to investigate the direction of genetic change in wild populations and quantify introgression, a pool of 375 farmed salmon, sampled over a five year period from 48 cages located on 35 farms spanning the Norwegian coastline were included in the present study. While this pool of farmed salmon does not necessarily accurately represent the allele frequencies of escapees entering all of these rivers for the entire study period, they clearly permitted elucidation of the direction of the genetic changes in wild populations (Figures 3 and 4). Furthermore, in the populations Opo and Vosso, which showed the highest estimated introgression rates, the observed direction of genetic change in relation to this pool of farmed salmon, and the simulated direction in relation to gene-flow from the pool of farmed salmon displayed almost perfect overlap (Figure 5). Thus, these results strongly indicate that this approach is valid. Nevertheless, it is suggested that a more accurate estimation of introgression of farmed salmon could be achieved if samples of farmed fish entering each specific river were collected together with samples of adult wild fish. If this was conducted yearly, together with sampling offspring from the subsequent generation at different life history stages (Figure 7), this would provide the most robust estimations of the allele frequencies for the native and intruding farmed fish. This cohort-based sampling could then be combined with the ABC and fixed migration simulations presented here to quantify introgression per year and thereafter per generation.

The offspring of farmed salmon display genetically-based lower survival in the natural environment compared to wild salmon [66-69]. Thus, it is likely that the frequency of farmed and hybrid salmon in a cohort will decrease with time. While this was not tested here, it is noteworthy that the two populations displaying the greatest introgression levels, i.e., Opo and Vosso, were represented by juvenile as opposed to adult samples. It is therefore possible that the level of farmed salmon introgression in these two populations would have been less if the contemporary samples for each of these rivers were adults. The cohort-based design (Figure 7) would permit addressing this issue, and potentially provide estimations in strength of selection against farmed fish in the different rivers, for the different life-history stages.

### Implications for management

The direct translation of molecular genetic data into aquaculture or fisheries management and regulation has not been without its challenges [70,71]. Good examples include identification of the farm of origin for escapees [38,41], poaching from protected populations [72], fishing competition fraud [73], and regulation of harvest at the individual [74] and population level [75]. Nevertheless, there is still a need to improve the translation of molecular genetic data into a “currency” that governing bodies can implement if these techniques are to find a routine place in the management of aquaculture and fisheries resources. While it is acknowledged that the methods for quantification of introgression presented here are still in need of further refinement, they provide management authorities with the first multiple-population estimations of introgression of farmed salmon, an essential early step in risk assessment [23]. Nevertheless, other important management issues, such as what early warning indicators for introgression exist, and what are the biological consequences of introgression, remain.

One of the limitations in using genetics techniques to quantify introgression of farmed salmon in native populations is that fact that it can only be used to validate introgression. While this is important, the obvious management target for mitigation prior to introgression is the frequency of farmed salmon in the native population. However, the correlation between some of the genetic parameters investigated here and the frequency of farmed salmon observed in these populations was at best modest. This is consistent with observations from a previous study [21], and is likely to be associated with the fact that there are large gaps in the data reporting the numbers of escapees in rivers, and that the density of the native population appears linked with resilience of the population to introgression. The implications of this for managers is that monitoring the frequency of farmed salmon in wild populations, or modeling genetic changes in the populations based upon the observed frequency of escapees [53,76], will not provide an accurate estimation of introgression for all populations.

There are widespread concerns regarding the genetic integrity and long-term fitness of native populations where large numbers of escapees have been observed [13-15]. However, while introgression of farmed escaped salmon has been documented in rivers in several countries, biological consequences of introgression has thus far not been reported for any wild Atlantic salmon population. This provides several challenges for managers. At what level should acceptable thresholds be set in the absence of fitness-consequence data in wild populations? And if a very low management threshold is set (for example <5% introgression), will the analytical methods and genetic markers available today, or in the near future, be able to accurately quantify introgression at such low levels? Given that there have been major advances in the use of sterile triploid Atlantic salmon for the commercial aquaculture, and 100% containment of aquaculture fish is ultimately unrealistic, management authorities should consider increasing efforts to convert the industry over to the use of sterile fish.

## Conclusion

This study is the first to quantify cumulative introgression of farmed salmon in any native Atlantic salmon population. Based upon ABC and fixed migration estimations, it has been demonstrated that introgression of farmed salmon in wild Norwegian Atlantic salmon populations has been population-specific, ranging from no detectable impact in some populations to strong introgression in others. Furthermore, where populations displayed clear temporal genetic changes, they all became more similar to a pool of farmed salmon. While the level of farmed salmon introgression was partially correlated with the frequency of escapees observed in the population, it is concluded that other mechanisms, such as the density of the recipient native population, is likely to influence the relative success of farmed fish. These data provide policy makers with unique information to address the influence of farmed escaped salmon on native populations.

## Competing interests

The authors declare that they have no competing interests.

## Authors’ contributions

KAG, ØS, FB and VW conceived and designed the study. MK conducted SNP genotyping. KAG, CP, FB and VW conducted statistic analyses. KAG and FB conceived and conducted the ABC and fixed migration computations to quantify introgression respectively. KAG wrote the first draft of the paper and coordinated the study. All authors contributed to and approved the final version of the manuscript.

## Supplementary Material

Additional file 1: Table S1 Characteristics of the 20 Atlantic salmon rivers including catch statistics and observed numbers of escapees. Click here for file

Additional file 2: Table S2 Technical details for the genetic markers used in the present study. Click here for file

Additional file 3: Table S3 Population genetic summary statistics for all wild samples and the pool of farmed salmon. Click here for file

Additional file 4: Table S4 Effective population size for all samples. Click here for file

Additional file 5: Figure S1 Distribution of pair-wise F_ST_ values between a 100 randomly selected farmed salmon vs. 100 randomly selected wild salmon using 47d and 25r. Click here for file

Additional file 6: Figure S2 Relationships between within-river genetic change as measured by 47d, 25r and 22 microsatellelites from previously published data. Click here for file

Additional file 7: Table S5 Bayesian clustering analysis for each population. Click here for file

Additional file 8: Figure S3 Percent exclusion of the 375 farmed salmon from each populations’s historic (blue–left bar) and contemporary (red–right bar) sample. Click here for file

Additional file 9: Figure S4 Principal component analysis for each geographic region based upon 47d and 25r. Click here for file

Additional file 10: Table S6 Matrix of pair-wise F_ST_ values among all samples included in the present study. Click here for file

Additional file 11: Figure S5 Principal compont analysis for the historical and contemporary samples for all 20 rivers and the group of farmed salmon based upon 47d and 25r. Click here for file
