# Supplementary material for: Atlantic salmon populations invaded by farmed escapees: quantifying genetic introgression with a Bayesian approach and SNPs
Source: BMC Genet. 2013 Aug 23;14:74. doi: 10.1186/1471-2156-14-74 (PMC3765417; doi:10.1186/1471-2156-14-74)
Supplement: Additional file 1: Table S1 — Characteristics of the 20 Atlantic salmon rivers including catch statistics and observed numbers of escapees. [file 1471-2156-14-74-S1.doc]

**Atlantic salmon populations invaded by farmed escapees: quantifying genetic introgression with a Bayesian approach and SNPs**

**Table S1. Characteristics of the 20 Atlantic salmon rivers including catch statistics and numbers of escapees.**

| Population |  | | Farmed escapees in the river | | |  | River characteristics | | | | | | | |
| --- | --- | --- | --- | --- | --- | --- | --- | --- | --- | --- | --- | --- | --- | --- |
|  | | Years counted | | Unweighted mean* (Range) | Weighted mean** |  | Local stocking? | 2010 catch (kg) | 2010 catch (n) | 1990 catch (kg) | 1990 catch (n) | Anadromous area (km^2^) | Conservation attainment (2007-2010) |  |
| Neiden | | 1 | | 12% | 2% |  | No | 4.907** | 1390 | 7099 | NA | 21.4 | 98% |  |
| V. Jakobselv | | 18 | | 30% (3-65) | 20% |  | No | 7.127 | 2283 | 1008 | 272 | 15.4 | 322% |  |
| Alta | | 15 | | 6% (0-22) | 5% |  | M(S) | 15.865 | 3403 | 9959 | 1953 | 57.0 | 228% |  |
| Reisa | | 12 | | 31% (0-100) | 5% |  | L | 7.280 | 1324 | 3044 | 585 | 53.0 | 177% |  |
| Målselv | | 15 | | 16% (4-36) | 8% |  | L | 11.614 | 2362 | 4992 | 908 | 20.0 | 249% |  |
| Roksdalsvass. | | 19 | | 7% (0-47) | 3% |  | No | 1.317 | 556 | NA | NA | 3.3 | 130% |  |
| Namsen | | 21 | | 27% (10-59) | 11% |  | L(A) | 20.360 | 4818 | 32075 | 8019 | 190.7 | 188% |  |
| Surna | | 7 | | 28% (0-56) | 14% |  | H(S+F) | 7.320 | 1364 | 7750 | 2348 | 35.1 | 136% |  |
| Eira | | 7 | | 16% (0-44) | 17% |  | H(S+P) | 2206 | 549 | 580 | NA | 7.0 | 119% |  |
| Bondalselva | | 10 | | 27% (0-83) | 17% |  | L(A) | 521 | 175 | 7500 | 2143 | 2.1 | 124% |  |
| Ørstaelva | | 15 | | 41% (8-78) | 22% |  | M(A) | 1.375 | 502 | 4040 | 1616 | 4.9 | 60% |  |
| GaulaSF | | 13 | | 31% (4-65) | 17% |  | M(A+E) | 891 | 300 | 2071 | 628 | 10.5 | 144% |  |
| Lærdalselva | | 4 | | 2% (0-2) | 4% |  | H(F) | Banned* | NA | 4371 | 599 | 18.2 | NA |  |
| Vosso | | 14 | | 45% (0-71) | 29% |  | H(S+P) | Banned*** | NA | 880 | 91 | 15.3 | NA |  |
| Loneelva | | 16 | | 8% (0-26) | 7% |  | M(A+F) | 244 | 107 | 363 | 214 | 0.4 | 133% |  |
| Opo | | 2 | | 50% (0-100) | 89% |  | L(F+S) | Banned*** | NA | 612 | 146 | 5.8 | NA |  |
| Etne | | 19 | | 57% (0-100) | 35% |  | L(E+S) | Banned*** | NA | 7778 | 2431 | 3.7 | 156% |  |
| Figgjo | | 14 | | 9% (0-28) | 9% |  | L(A+E) | 4393 | 1466 | 7326 | 3330 | 5.4 | 175% |  |
| Numedalslågen | | 15 | | 7% (0-50) | 5% |  | L(A) | 7.729 | 1695 | 8791 | 2442 | 79.4 | 93% |  |
| Berbyelva | | 6 | | 4% (0-11) | 2% |  | L | 1134 | 181 | 304 | 74 | 3.3 | 582% |  |
|  | |  | |  |  |  |  |  |  |  |  |  |  |  |

Years counted = numbers of years in which farmed salmon were counted in the river, % of farmed salmon = the mean percent of farmed salmon observed in these populations based upon the unweighted mean = average percentage of farmed salmon in spawning population in the period 1989-2009 , weighed mean = weighted average percentage of farmed salmon in the population combining data from both sports-fishing and spawning population samples ; range for the unweighted mean refers to the lowest and maximum percentages of farmed salmon observed in the spawning populations (this also includes recordings with very low numbers of observations in some years ). Local stocking history and river catch in 2010 statistics Norway [www.ssb.no](http://www.ssb.no/), and 1990 ; Na = not available. * = treated against *Gyrodactylus salaris*; ** = Norwegian zone; *** = population collapse or strongly reduced; smolt and parr stocking activity: <5000 : Low; 5-15000: Medium; >15000: High (eggs, alevins and fry converted to smolt numbers by calculating 10% survival); anadromous area available to smolts , and conservation attainment which is the average attainment of the conservation limit for each specific river as defined by the numbers of female salmon left in the river after fishing mortality in relation to the number of eggs required to achieve the rivers estimated carrying capacity .

References

Anon., 1992. Fiske og oppdrett av laks mv. (Fishing and rearing of salmon etc.). Noregs ofisielle statistikk C56. (In Norwegian).

Anon., 2010. Vedleggsrapport merd vurdering av måloppnåelse for de enkelte bestandene. Rapport fra Vitenskapelig råd for Lakseforvaltning nr 2b. (In Norwegian).

Anon., 2011. Status for norske laksebestander i 2011. Rapport fra vitenskapelig råd for lakseforvaltning nr 3, 285s. (In Norwegian).

Diserud, O.H., Fiske, P. and Hindar, K., 2012. Forslag til kategorisering av laksebestander som er påvirket av rømt oppdrettslaks. NINA Rapport 782 32s. (In Norwegian).

Fiske, P., Lund, R.A., Østborg, G.M. and Fløystad, L., 2001. Rømt oppdrettslaks i sjø- og elvefisket i årene 1989-2000. NINA oppdresgsmelding 704: 1-26. (In Norwegian).
