# Supplementary material for: Atlantic salmon populations invaded by farmed escapees: quantifying genetic introgression with a Bayesian approach and SNPs
Source: BMC Genet. 2013 Aug 23;14:74. doi: 10.1186/1471-2156-14-74 (PMC3765417; doi:10.1186/1471-2156-14-74)
Supplement: Additional file 5: Figure S1 — Distribution of pair-wise FST values between a 100 randomly selected farmed salmon vs. 100 randomly selected wild salmon using 47d and 25r. [file 1471-2156-14-74-S5.doc]

**Atlantic salmon populations invaded by farmed escapees: quantifying genetic introgression with a Bayesian approach and SNPs**

**Figure S1. Distribution of pair-wise F_ST_ values between a 100 randomly selected farmed salmon vs. 100 randomly selected wild, 100 randomly selected farmed salmon vs. 100 randomly selected farmed salmon, and 100 randomly selected wild salmon vs. 100 randomly selected wild salmon. This was computed using 47d (top) and 25r (bottom). All distributions created from 1000 bootstraps.**
