# Supplementary material for: Atlantic salmon populations invaded by farmed escapees: quantifying genetic introgression with a Bayesian approach and SNPs
Source: BMC Genet. 2013 Aug 23;14:74. doi: 10.1186/1471-2156-14-74 (PMC3765417; doi:10.1186/1471-2156-14-74)
Supplement: Additional file 6: Figure S2 — Relationships between within-river genetic change as measured by 47d, 25r and 22 microsatellelites from previously published data. [file 1471-2156-14-74-S6.ppt]

## Slide 1
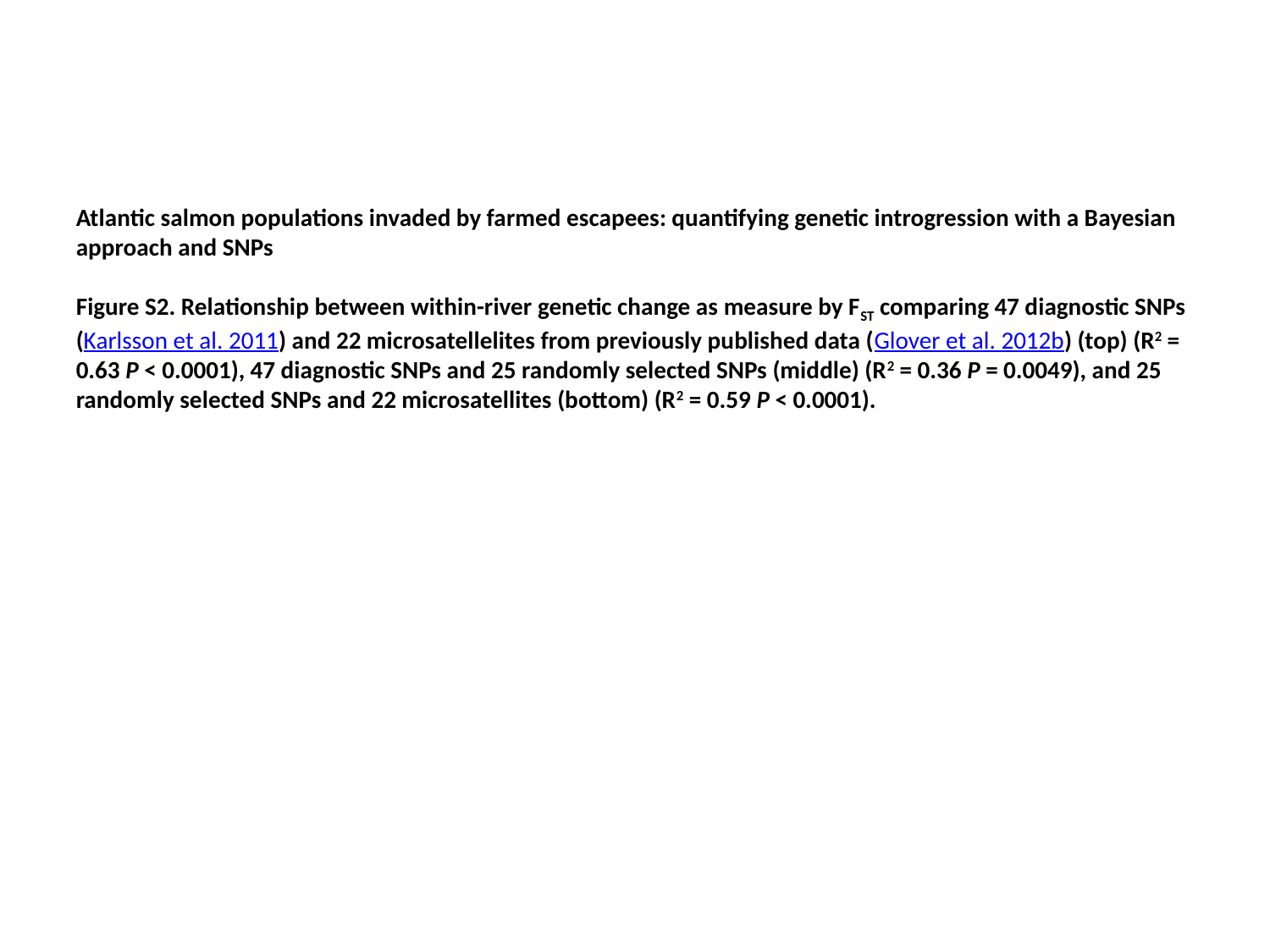

# Atlantic salmon populations invaded by farmed escapees: quantifying genetic introgression with a Bayesian approach and SNPs Figure S2. Relationship between within-river genetic change as measure by FST comparing 47 diagnostic SNPs (Karlsson et al. 2011) and 22 microsatellelites from previously published data (Glover et al. 2012b) (top) (R2 = 0.63 P < 0.0001), 47 diagnostic SNPs and 25 randomly selected SNPs (middle) (R2 = 0.36 P = 0.0049), and 25 randomly selected SNPs and 22 microsatellites (bottom) (R2 = 0.59 P < 0.0001).

## Slide 2
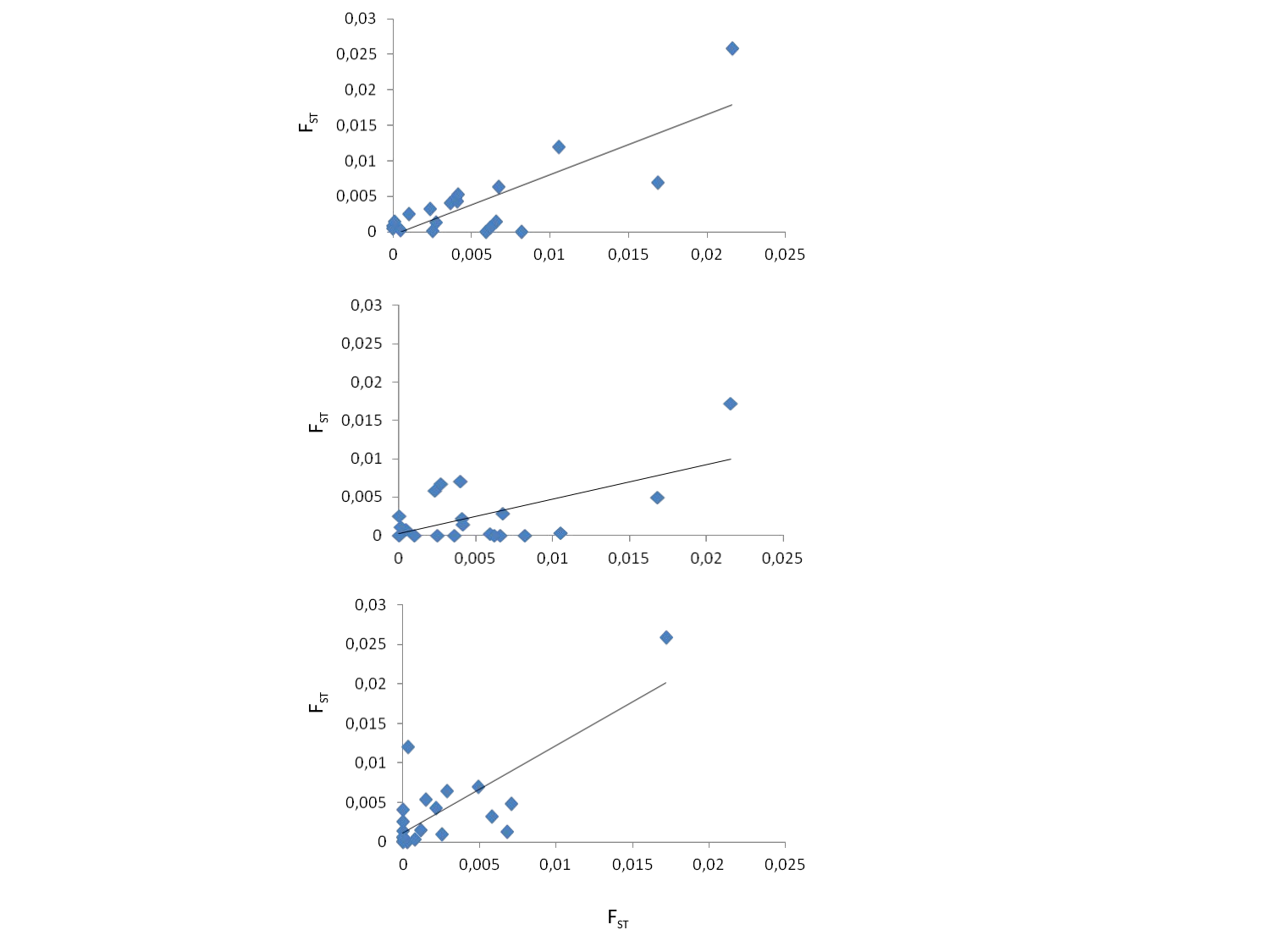

FST
FST
FST
FST
