# Supplementary material for: Atlantic salmon populations invaded by farmed escapees: quantifying genetic introgression with a Bayesian approach and SNPs
Source: BMC Genet. 2013 Aug 23;14:74. doi: 10.1186/1471-2156-14-74 (PMC3765417; doi:10.1186/1471-2156-14-74)
Supplement: Additional file 7: Table S5 — Bayesian clustering analysis for each population. [file 1471-2156-14-74-S7.doc]

Atlantic salmon populations invaded by farmed escapees: quantifying genetic introgression with a Bayesian approach and SNPs

**Table S5. Admixture analysis of 20 Atlantic salmon populations distributed throughout Norway.**

**Notes to figures.**

Sample size (N) and time span for historic (H), and contemporary sampling (C), ordered in the barplot with increasing numbers. Inferred ancestry of individuals was calculated with STRUCTURE v.2.3.4 for a data set of 72 microsatellite and 47 SNP loci under a model assuming admixture and correlated allele frequencies without using population information. Five runs with a burn-in period consisting of 250000 replications and a run length of 500000 Markov chain Monte Carlo (MCMC) iterations were performed for a number of clusters ranging from K 1 to 5. Admixture plots for K=2 is plotted for each population.

| **River** | **Sample** | **N** | **Time span** | **STRUCTURE barplot ALL 72 MARKERS** | **STRUCTURE barplot 47 DIAGNOSTIC MARKERS** |
| --- | --- | --- | --- | --- | --- |
| Neiden | NeidenH | 70 | 1979-1982 | 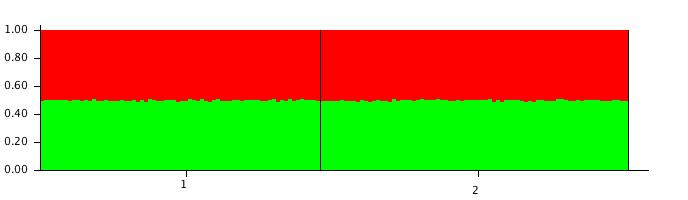 | 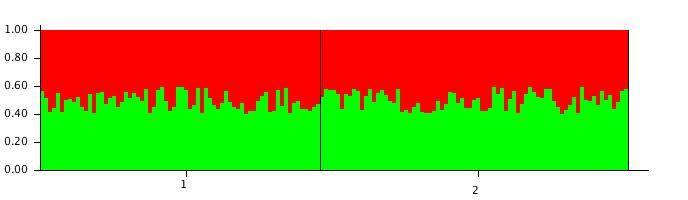 |
|  | NeidenC | 77 | 2009 |  |  |
| Vestre Jakobselva | V.JakobsH | 92 | 1989-1990-1991 | 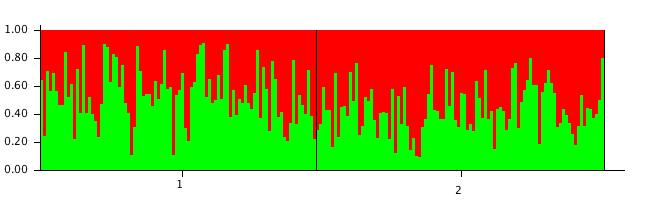 | 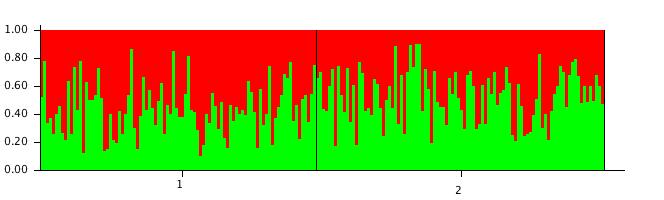 |
|  | V.JakobsC | 96 | 2007-2008 |  |  |

| **River** | **Sample** | **N** | **Time span** | **STRUCTURE barplot ALL 72 MARKERS** | **STRUCTURE barplot 47 DIAGNOSTIC MARKERS** |
| --- | --- | --- | --- | --- | --- |
| Alta | AltaH | 39 | 1988-1989-1990 | 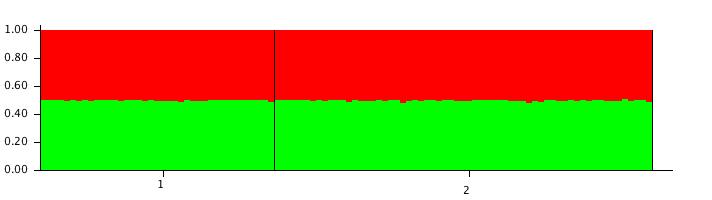 | 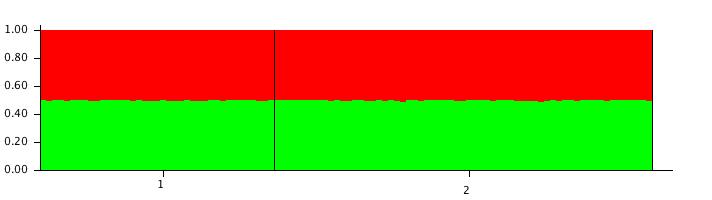 |
|  | AltaC | 63 | 2005-2007 |  |  |
| Reisa | ReisaH | 44 | 1986-1987-1988-1989-1990-1991 | 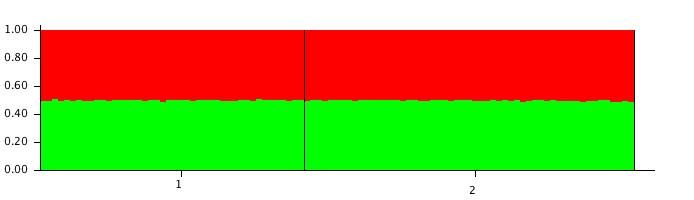 | 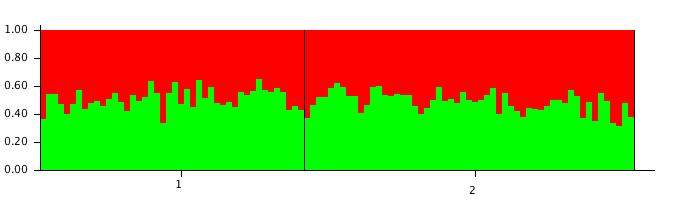 |
|  | ReisaC | 55 | 2006 |  |  |
| Målselva | MålselvH | 39 | 1986-1987-1988 | 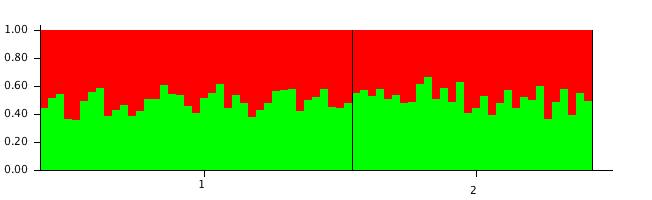 | 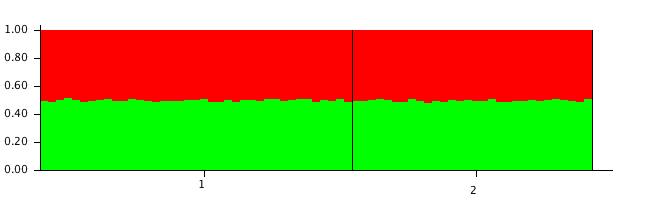 |
|  | MålselvC | 30 | 2008 |  |  |
| Roksdalsvassdragget | RoksdalsH | 31 | 1987-1988-1989-1990-1991-1992-1993 | 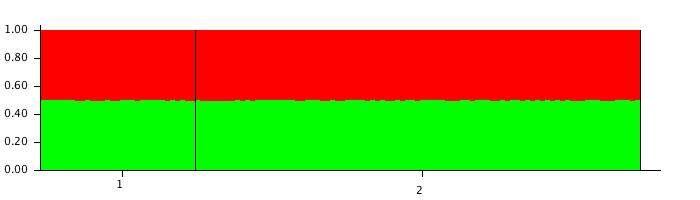 | 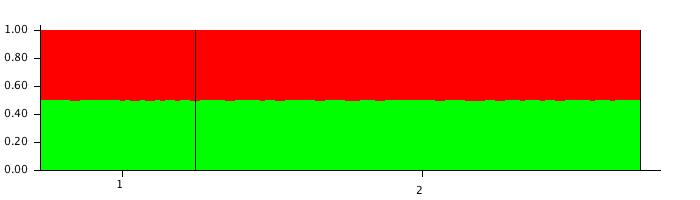 |
|  | RoksdalsC | 89 | 2008 |  |  |
| Namsen | NamsenH | 74 | 1977 | 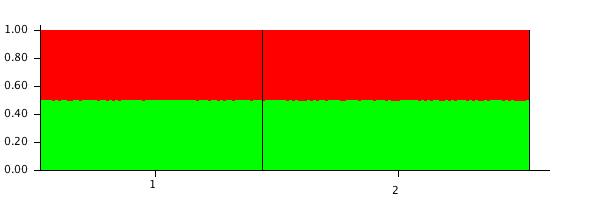 | 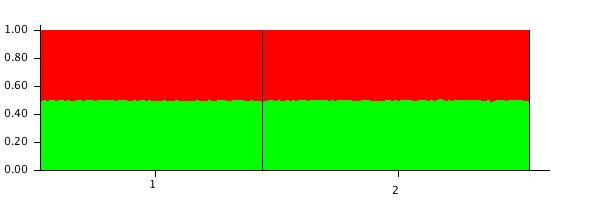 |
|  | NamsenC | 89 | 2008 |  |  |

| **River** | **Sample** | **N** | **Time span** | **STRUCTURE barplot ALL 72 MARKERS** | **STRUCTURE barplot 47 DIAGNOSTIC MARKERS** |
| --- | --- | --- | --- | --- | --- |
| Surna | SurnaH | 23 | 1986-1987-1988-1989 | 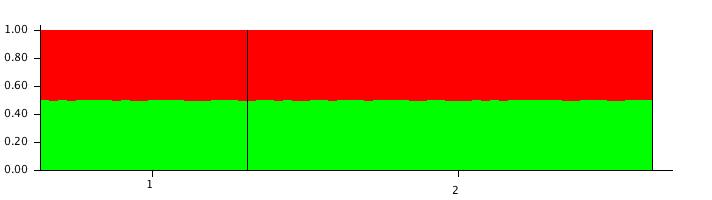 | 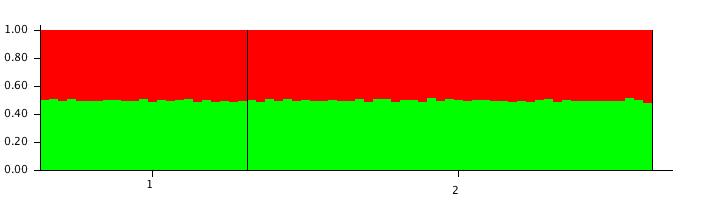 |
|  | SurnaC | 45 | 2005-2006-2007-2008 |  |  |
| Eira | EiraH | 31 | 1986-1987-1988-1992-1994 | 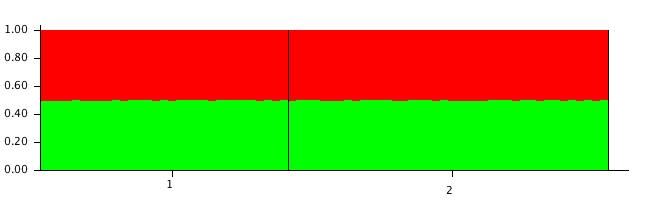 | 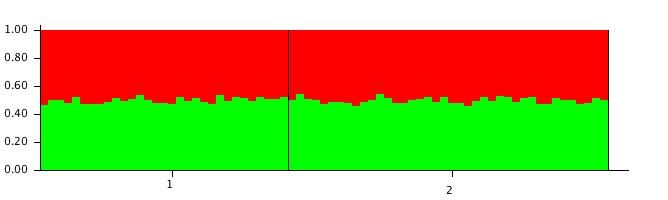 |
|  | EiraC | 40 | 2005-2006-2007-2008 |  |  |
| Bondalselva | BondalsH | 39 | 1986-1987-1988 | 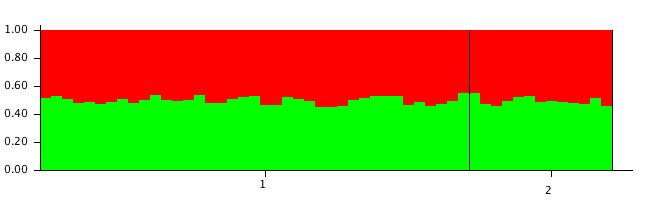 | 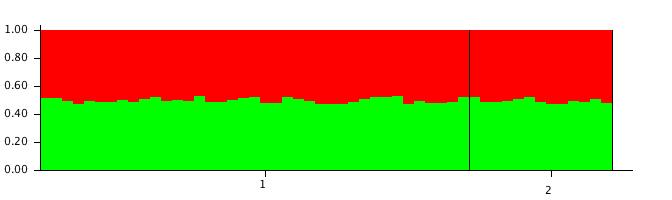 |
|  | BondalsC | 13 | 2007 |  |  |
| Ørstaelva | ØrstaH | 38 | 1986-1987-1988-1989 | 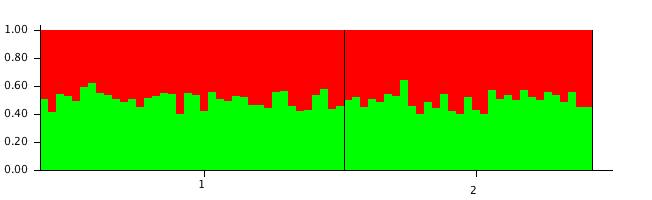 | 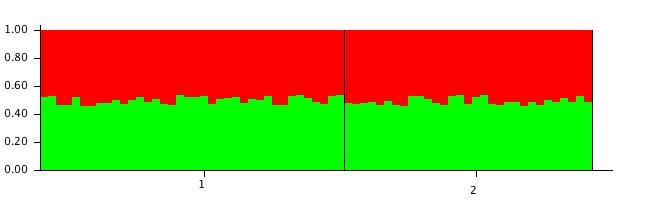 |
|  | ØrstaC | 31 | 2006-2008 |  |  |

| **River** | **Sample** | **N** | **Time span** | **STRUCTURE barplot ALL 72 MARKERS** | **STRUCTURE barplot 47 DIAGNOSTIC MARKERS** |
| --- | --- | --- | --- | --- | --- |
| Gaula SF | Gaula SFH | 35 | 1987-1988-1989-1990-1991-1992-1993 | 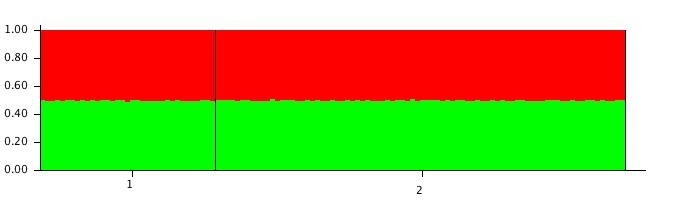 | 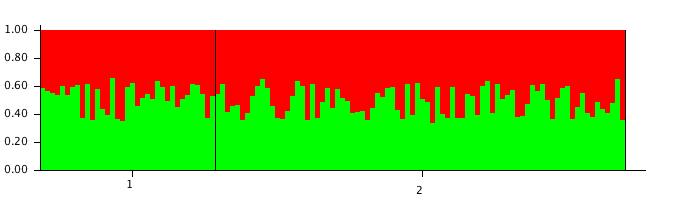 |
|  | Gaula SFC | 82 | 2006-2008 |  |  |
| Lærdalselva | LærdalsH | 90 | 1973 | 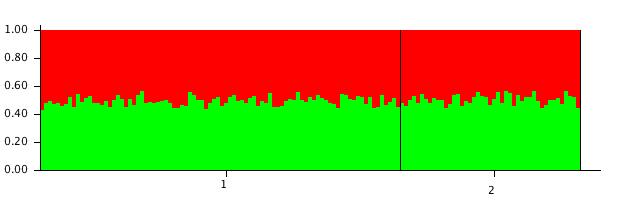 | 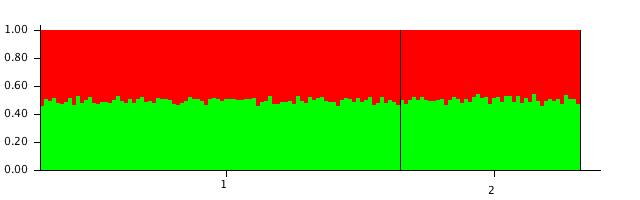 |
|  | LærdalsC | 45 | 2005-2006-2007-2008 |  |  |
| Vosso | VossoH | 45 | 1980 | 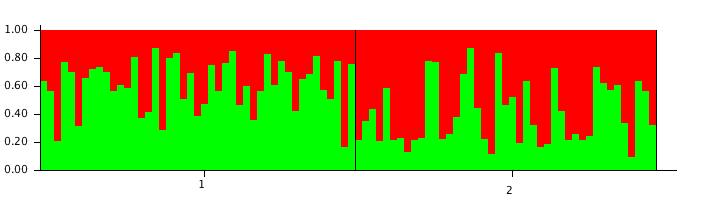 | 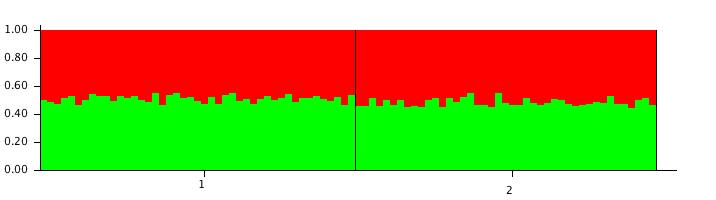 |
|  | VossoC | 43 | 2008 |  |  |
| Loneelva | LoneH | 59 | 1986-1987-1988-1989-1993 | 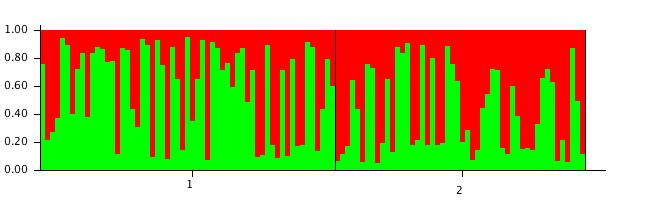 | 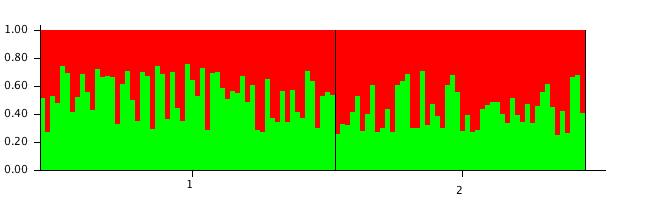 |
|  | LoneC | 50 | 2001-2005-2006-2007 |  |  |

| **River** | **Sample** | **N** | **Time span** | | **STRUCTURE barplot ALL 72 MARKERS** | | **STRUCTURE barplot 47 DIAGNOSTIC MARKERS** | |
| --- | --- | --- | --- | --- | --- | --- | --- | --- |
| Opo | OpoH | 60 | | 1971-1973 | | 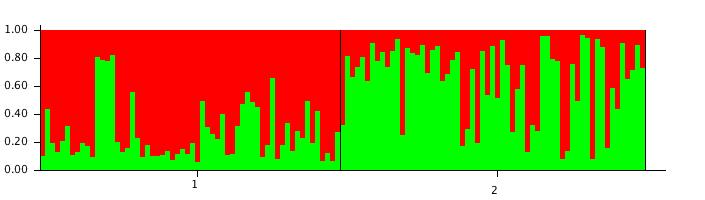 | | 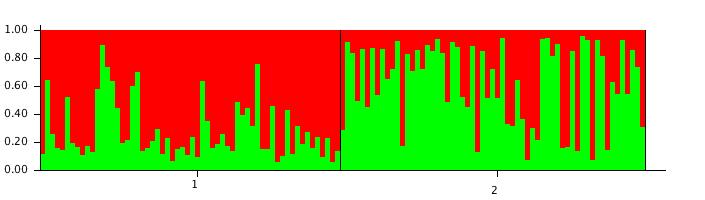 |
|  | OpoC | 61 | | 2010 | |  |  |  |
| Etne | EtneH | 72 | 1983 | | 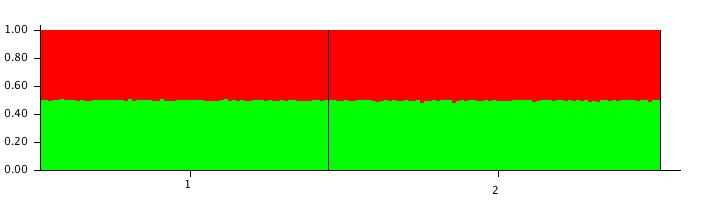 | | 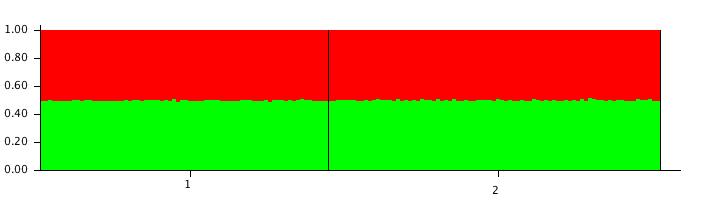 | |
|  | EtneC | 83 | 2006-2007-2008 | |  |  |  |  |
| Figgjo | FiggjoH | 51 | 1972-1973-1974-1975 | | 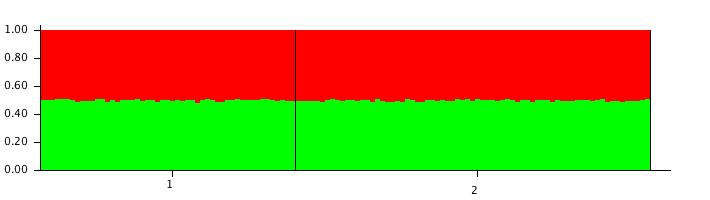 | | 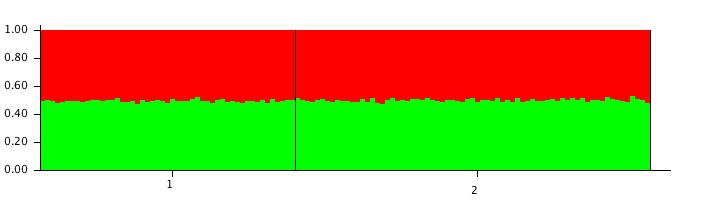 | |
|  | FiggjoC | 71 | 2006 | |  |  |  |  |
| Numedalslågen | NumedalsH | 42 | 1989-1990-1991-1992-1993 | | **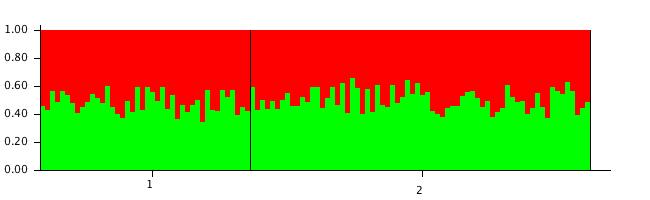** | | 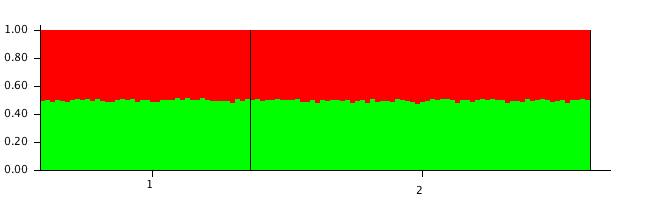 | |
|  | NumedalsC | 68 | 2007-2008 | |  |  |  |  |

| **River** | **Sample** | **N** | **Time span** | **STRUCTURE barplot ALL 72 MARKERS** | **STRUCTURE barplot 47 DIAGNOSTIC MARKERS** |
| --- | --- | --- | --- | --- | --- |
| Berbyelva | BerbyelvaH | 44 | 1988-1989-1990-1991-1992-1993 | **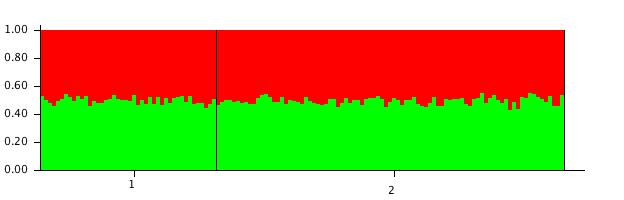** | 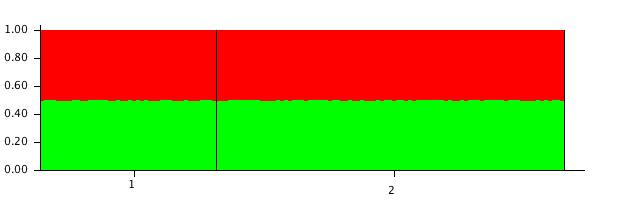 |
|  | BerbyelvaC | 87 | 2007-2008 |  |  |
|  |  | **3049** | **1971-2010** |  |  |
