# Supplementary material for: Atlantic salmon populations invaded by farmed escapees: quantifying genetic introgression with a Bayesian approach and SNPs
Source: BMC Genet. 2013 Aug 23;14:74. doi: 10.1186/1471-2156-14-74 (PMC3765417; doi:10.1186/1471-2156-14-74)
Supplement: Additional file 8: Figure S3 — Percent exclusion of the 375 farmed salmon from each populations’s historic (blue–left bar) and contemporary (red–right bar) sample. [file 1471-2156-14-74-S8.doc]

**Atlantic salmon populations invaded by farmed escapees: quantifying genetic introgression with a Bayesian approach and SNPs**

**Figure S3. Percent exclusion of the 375 farmed salmon from each populations´s historic (blue – left bar) and contemporary (red – right bar) sample, computed using all 72 SNPs (Top), 47d (middle) and 25r (bottom). X axis = population name, Y axis = percentage of farmed salmon excluded at at *P* < 0.001.**

**
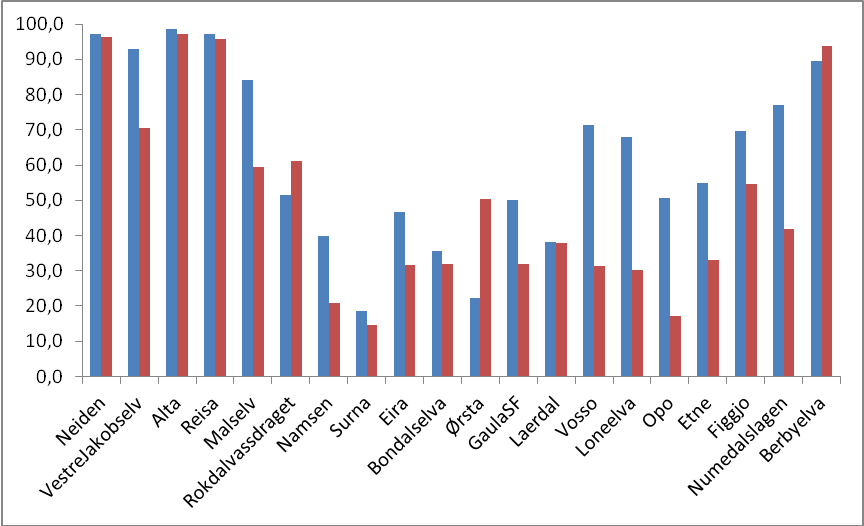
**

**
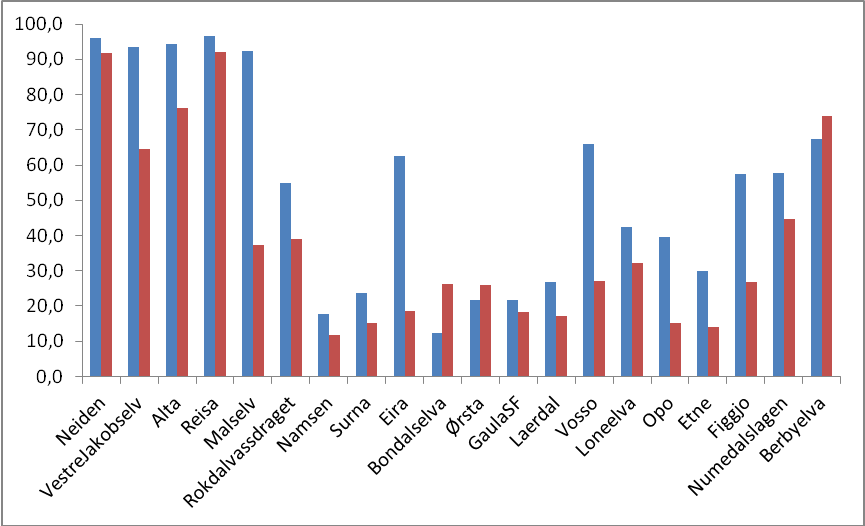
**

**
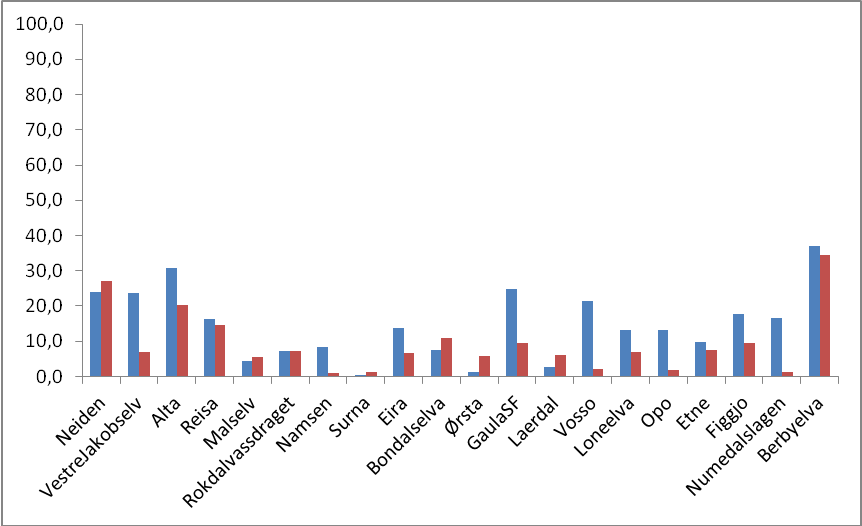
**
